# Targeted Nanocarrier-Based Drug Delivery Strategies for Improving the Therapeutic Efficacy of PARP Inhibitors against Ovarian Cancer

**DOI:** 10.3390/ijms25158304

**Published:** 2024-07-30

**Authors:** Patrycja Gralewska, Arkadiusz Gajek, Agnieszka Marczak, Aneta Rogalska

**Affiliations:** Department of Medical Biophysics, Institute of Biophysics, Faculty of Biology and Environmental Protection, University of Lodz, Pomorska 141/143, 90–236 Lodz, Poland; patrycja.gralewska@biol.uni.lodz.pl (P.G.); arkadiusz.gajek@biol.uni.lodz.pl (A.G.); agnieszka.marczak@biol.uni.lodz.pl (A.M.)

**Keywords:** PARPi, targeted nanotherapy, nanoparticles, active targeting, ovarian cancer

## Abstract

The current focus of ovarian cancer (OC) research is the improvement of treatment options through maximising drug effectiveness. OC remains the fifth leading cause of cancer-induced mortality in women worldwide. In recent years, nanotechnology has revolutionised drug delivery systems. Nanoparticles may be utilised as carriers in gene therapy or to overcome the problem of drug resistance in tumours by limiting the number of free drugs in circulation and thereby minimising undesired adverse effects. Cell surface receptors, such as human epidermal growth factor 2 (HER2), folic acid (FA) receptors, CD44 (also referred to as homing cell adhesion molecule, HCAM), and vascular endothelial growth factor (VEGF) are highly expressed in ovarian cancer cells. Generation of active targeting nanoparticles involves modification with ligands that recognise cell surface receptors and thereby promote internalisation by cancer cells. Several poly(ADP-ribose) polymerase (PARP) inhibitors (PARPi) are currently used for the treatment of high-grade serous ovarian carcinomas (HGSOC) or platinum-sensitive relapsed OC. However, PARP resistance and poor drug bioavailability are common challenges, highlighting the urgent need to develop novel, effective strategies for ovarian cancer treatment. This review evaluates the utility of nanoparticles in ovarian cancer therapy, with a specific focus on targeted approaches and the use of PARPi nanocarriers to optimise treatment outcomes.

## 1. Introduction

Ovarian cancer (OC) is the third most common gynaecological cancer and the fifth leading cause of cancer-related mortality in women [1,2]. The primary factor contributing to poor prognosis is the presence of advanced disease in more than 70% of patients, resulting in 5-year overall survival rates ranging from 15% to 45%. On the other hand, the survival rate for patients with stage I cancer, in which the tumour is restricted to the ovaries only, is >90% [3,4]. The mainstay of standardised clinical treatment for ovarian cancer is platinum- and taxane-based chemotherapy after cytoreductive surgery. However, patients with *CCNE1* amplification display intrinsic resistance to platinum-based chemotherapy [5]. The progressive development of poly(ADP-ribose) polymerase (PARP) inhibitors (PARPi) has revolutionised the management of patients with high-grade serous ovarian carcinoma (HGSOC). Around 50% of HGSOC cases exhibit defects in the homologous recombination (HR) pathway [6], which is typically characterised by germline or somatic *BRCA1* or *BRCA2* mutations that represent ~20% of cases [7]. The primary mechanism of action of PARPi against *BRCA*-mutated HGSOC is synthetic lethality. Without PARP activity, single-strand breaks (SSBs) cannot be repaired efficiently, leading to double-strand breaks (DSBs) and cell death via apoptosis due to mitotic catastrophe [8,9].

Chemotherapy often involves the use of a single drug to inhibit or control cancer cells. However, the effectiveness of monotherapy is commonly compromised, mainly due to the development of multidrug resistance and undesirable side effects [10]. One of the adverse effects of chemotherapy is the destruction of normal cells, which weakens the immune system. Traditional chemotherapy using cytotoxic drugs or targeted molecular inhibitors can fail when ovarian cancer cells acquire resistance, undergo metastasis, or patients no longer tolerate treatment due to severe adverse effects. Furthermore, PARPi compounds are mainly administered orally, which reduces drug bioavailability due to first-pass metabolism and relatively low drug accumulation at the tumour tissue site [11].

**Figure 1:**
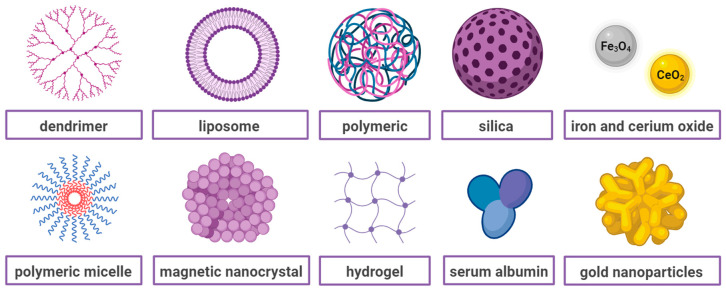
A simplified graphical illustration of various types of nanoparticles used in ovarian cancer treatment.

The main objective of current ovarian cancer research is to improve therapeutic options for maximising drug effectiveness. Nanotechnology serves as a powerful tool in the development of novel approaches for the treatment of multiple diseases, including ovarian cancer. Over recent years, several nanoformulations have been developed as drug delivery systems (DDS) for the treatment of OC, including liposomes, polymeric micelles, hydrogels, nanoemulsions, nanosuspensions, and other NPs (Figure 1). The benefits of nanoparticle systems are that they are highly stable, specific, efficient, and capable of producing both hydrophobic and hydrophilic drugs. Improved drug solubility and stability enhance the bioavailability and, thus, therapeutic efficacy of the drugs. Low-solubility drugs can be either enclosed in the hydrophobic interface of the nanocarrier or act as a carrier for drugs in the blood. Biocompatibility is another advantage of nanoparticles. They are intended and designed to be non-toxic, which reduces the risk of immune responses. Improved selectivity, hence reducing the side effects by concentrating the therapeutic agents in the tumour and not normal cells, has been achieved through the modification of nanoparticles with antibodies, fragments of antibodies, peptides, and growth factors that bind to specific receptors overexpressed in ovarian cancer cells [12,13]. Functionalisation with various ligands, specifically binding to markers on ovarian cancer cells, also provides a high degree of customisation for personalised medicine approaches. Moreover, the nanotheranostics approach allows the co-delivery of targeting ligands, drugs, and imaging agents, thereby simultaneously achieving therapeutic, diagnostic, and real-time traceable drug delivery objectives [14]. Enhanced imaging capabilities can lead to earlier detection of ovarian cancer, whereas real-time monitoring of treatment efficacy allows for adjustments in therapy as needed.

This review provides a concise summary of the use of various types of nanoparticles in ovarian cancer treatment, with a specific focus on targeted therapies and PARPi nanocarriers.

## 2. Types of Nanoparticles Employed for Ovarian Cancer Therapy

Solid tumours frequently exhibit an enhanced permeability and retention (EPR) effect, which is correlated with reduced lymphatic drainage, increased angiogenesis, and enhanced permeability of vessels [15]. This effect promotes the accumulation of nanoparticles within the tumour and significantly reduces off-target toxicity since normal tissues lack EPR [16], a process known as passive targeting.

**Figure 2:**
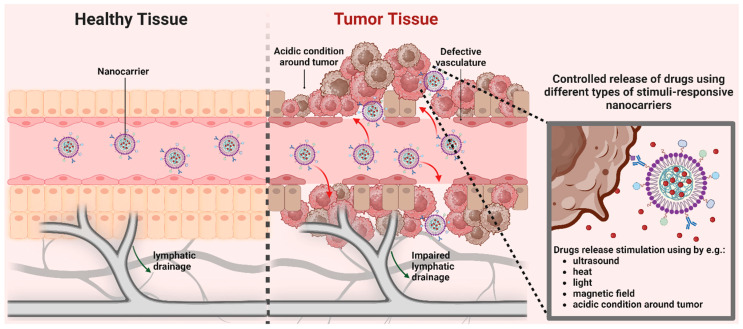
The controlled release of drugs from nanoparticles that respond to tumour tissue microenvironment.

The mechanism by which nanoparticles enter solid tumours, but not normal tissues, is more complicated. In addition to the passive entry of nanostructures through large gaps between endothelial cells, the tumour microenvironment (TM) is important in this process, particularly in hypoxia and acidic conditions. Additionally, immune cells in TM play an important role in the accumulation, retention, and distribution of nanodrugs inside the tumour. According to the mechanism by which the drug is ejected from the nanocarrier, the release of the drug can be divided into four main categories: diffusion, solvent, chemical reaction and stimuli-controlled. However, the release of drugs from carriers is affected by a number of factors, including the composition of the nanoparticle, the ratio of the components within the NPs, the physical and chemical interaction between components, and the preparation method [17]. Stimuli-responsive nanocarriers, activated by pH, redox, and light conditions, exhibit the potential to accelerate cancer therapy base on the immune cells [18,19]. The TM also shows several enzyme secretions consisting of MMPs, hyaluronidase, γ-glutamyl transpeptidase, and esterase with higher expression in tumours compared to normal tissues, which can be used in the drug release from nanoparticles in the nearby tumour cells [20,21]. The mechanisms of action of stimuli-responsive nanocarriers are shown in Figure 2.

**Figure 3:**
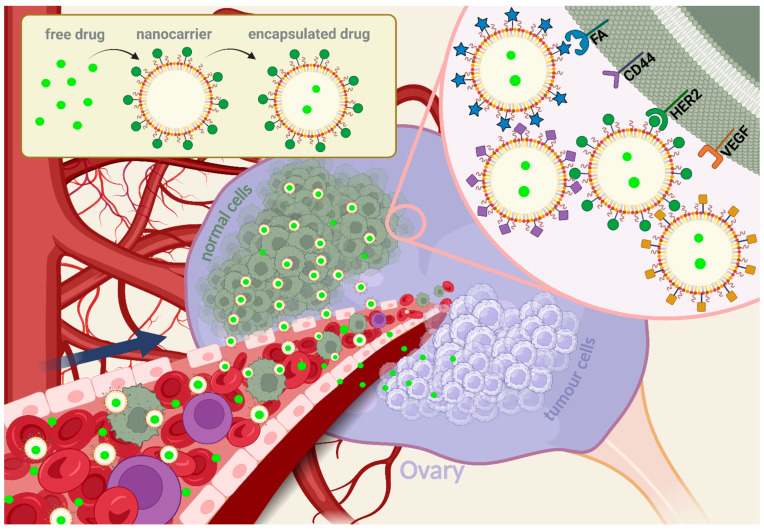
Schematic representation of the mechanism of action of ovarian cancer nanoparticles. Free drugs accumulate at both normal and tumour tissue sites, whereas drugs encapsulated in nanocarriers are located in cancer tissue using the EPR effect. Receptor-mediated active targeting promotes drug accumulation predominantly in the tumour tissue because of the specific ligands present on the surface, leading to improved selectiveness and therapeutic responses.

However, the rate of leakage from vessels is slow, and the drug may be metabolised or excreted before therapeutic levels are reached [22,23]. Active targeting is achieved by functionalisation of nanoparticles with targeted surface receptors that are highly expressed in tumour cells, such as human epidermal growth factor 2 (HER2), folic acid (FA), CD44 (also referred to as homing cell adhesion molecule, HCAM), or vascular endothelial growth factor (VEGF) in ovarian cancer, is a more specific strategy (Figure 3). This process takes advantage of aberrant expression of cell surface proteins, allows increased internalisation by cancer cells, and has been shown to bypass drug efflux by P-glycoprotein, also known as multidrug resistance protein 1 (P-gp or MDR1), thereby overcoming chemoresistance and improving the therapeutic response [24,25,26].

When it comes to the mode of drug delivery with nanocarriers, three different types can be distinguished: encapsulation, conjugation, and polyplex formation. Encapsulation takes place when the drugs are encapsulated within either the lipid bilayer or aqueous core of liposomes, within the biodegradable polymers, such as poly(lactic-co-glycolic acid) (PLGA), or within the internal cavities or branches of the dendrimers. This method provides controlled and sustained drug release [27]. Conjugation is when the drugs are covalently attached to the surface or the backbone of nanoparticles, which ensures stable attachment and controlled release upon reaching the target site or when targeting ligands (e.g., antibodies, peptides) are conjugated to the nanoparticle surface to facilitate active targeting of cancer cells overexpressing specific receptors [28]. Polyplexes are complexes between often cationic polymers (polyethylenimine (PEI), poly-L-lysine (PLL), poly(amidoamine) (PAMAM)) and nucleic acids (e.g., DNA, siRNA). Polyplexes protect genetic material from degradation and facilitate cellular uptake [29].

### 2.1. Plain Nanoparticles for Ovarian Cancer Treatment

**Figure 4:**
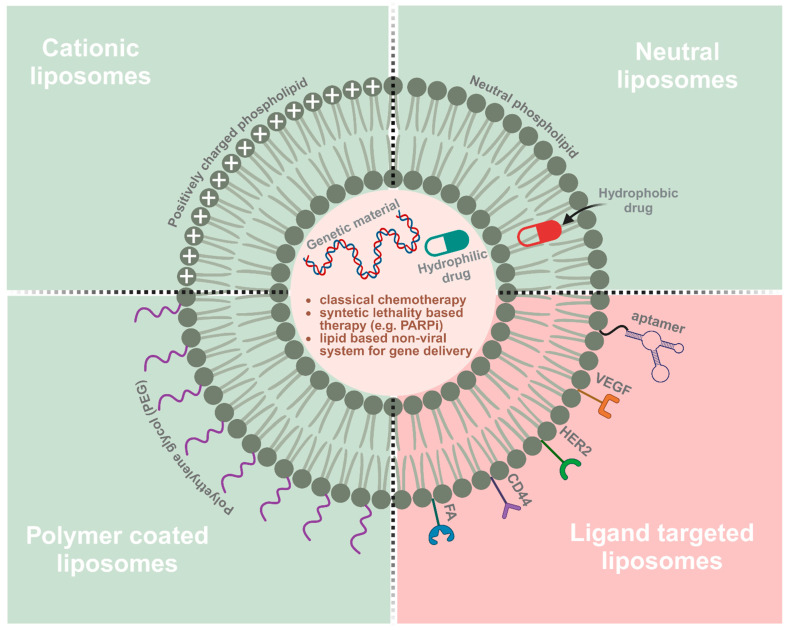
Types of liposomes used for chemotherapy and gene therapy in ovarian cancer: cationic liposomes; neutral liposomes; pegylated liposomes- PEG and ligands such as CD44, VEGFR, FR, or HER2 targeted liposomes.

Liposomes are the most extensively explored as nanocarriers for targeted drug delivery. These spherical phospholipid structures contain hydrophilic cores surrounded by a bilayer composed of amphiphilic lipid materials that facilitate the entrapment of both lipophilic and hydrophilic molecules. Hydrophobic compounds are inserted into the bilayer membrane, while hydrophilic compounds are captured in the aqueous interior [30,31]. Liposomes can also be divided into four major groups: neutral, cationic, pegylated, and ligand-targeted liposomes (Figure 4). Cationic liposomes can undergo stabilising electrostatic interactions with negatively charged nucleic acids and cell membranes to improve the transportation of large DNA or RNA. This structure limits the aggregation of liposomes in the blood [32]. Neutral liposomes reduce protein binding and uptake efficiency by endothelial cells to increase the plasma circulation time of the gene carrier. The lack of surface charge limits stability by increasing the aggregation of liposomes in the blood [33]. Leaky vasculature at the tumour site promotes the accumulation and activity of liposomes [34]. Polyethylene glycol (PEG)-modified liposomal doxorubicin (PLD) (Caelyx in Europe and Canada; Doxil in the USA) was the first nanodrug approved by the Food and Drug Administration (FDA) in 1995 for the treatment of ovarian cancer and multiple myeloma. PLD was approved by the FDA in 1999 and the European Medicines Agency (EMA) in 2000 as a single agent for advanced ovarian cancer patients who failed first-line platinum-based treatment [35,36]. Coating liposomes with synthetic polymers such as PEG is a crucial step in improving their properties as nanocarriers. The hydrophilic layer formed by the thick PEG head groups acts as a barrier to prevent interactions with plasma opsonins, thus increasing the longevity of the nanoparticles and prolonging their circulation time [37,38]. An earlier meta-analysis of randomised clinical trials demonstrated that PLD was equally as effective as other monotherapies and more tolerable in ovarian cancer patients. Furthermore, co-treatment with carboplatin and PLD was linked to longer progression-free survival (PFS), but similar overall survival (OS) relative to a combination of carboplatin and paclitaxel (PTX) used as standard ovarian cancer therapy. However, the drug combinations displayed distinct toxicity profiles. Patients receiving PLD and carboplatin experienced lower neuropathy, alopecia, and neutropenia, but had greater gastrointestinal toxicity, anaemia, and cutaneous toxicity [39]. Examples of clinical trials using PLD in combination with other agents are shown in Table 1.

An interesting nanocarrier, pH dual-responsive core cross-linked micelles (HCCL) based on a multi-functional amphiphilic linear-hyperbranched copolymer, utilising doxorubicin (DOX) was described by Tian et al. (2017). A self-condensing vinyl co-polymerisation of *tert*-butyl acrylate (*t*BA) and *p*-chloromethylstyrene (CMS) from a PEG-based initiator was used as a macroinitiator for the atom transfer radical polymerisation of *t*BA and N,N′-bis (acryloyl)cystamine (BACy) to fabricate a reduction responsive core-crossed linked micelles (CCL). Then, the HCLL micelles with DOX were prepared after hydrolysation of *t*BA units into acrylic acids one. However, a cytotoxicity assay performed on the ovarian cancer SKOV3 cell line showed that DOX-loaded HCCL micelles ability to cancer cell killing was similar to that of the free drug [40].

Another nanosystem is based on poly(butyl cyanoacrylate) (PBCA), a homopolymer of *n*-butyl-2-cyanoacrylate (BCA), and a representative member of the poly(alkyl cyanoacrylate) PACA family. The fundamental difference between PBCA and PLGA or poly(caprolactone) (PCL) is the lack of commercially available polymer matrices, which must be synthesised from the monomer before nanoparticle production [41]. The efficacy of both PEGylated and non-PEGylated PBCA-containing carboplatin were evaluated in A2780 ovarian cancer cell line resistant to cisplatin by Kanaani et al. (2017). The results showed decreased IC_50_ values for both nanodrugs compared to free drugs. The most significant difference was observed after 24 h of incubation, whereby IC_50_ values were ~26 µM for PEGylated nanodrugs relative to 54 µM for the free drug, and 38 µM for the non-PEGylated nano form relative to 61 µM for the free drug. After 72 h, IC_50_ values were more similar, specifically, 23 µM for PEGylated nano drug compared to 30 µM for the free form and 26 µM for non-PEGylated nano drug relative to 34 µM for the free drug [42].

Paclitaxel (PTX) is a frontline chemotherapeutic agent for ovarian cancer. A study by Shen et al. [43] compared the efficacy of liposomal PTX administered via intraperitoneal (IP) and intravenous (IV) delivery methods. Initially, researchers established a PTX-resistant ovarian cancer cell line, which was then inoculated into the peritoneal cavity of a mouse model. IP delivery of liposomal PTX achieved better results than IV delivery, which failed to show efficacy due to the inherent limitations of the EPR effect [43]. PTX was additionally encapsulated in a different lipid-dendrimer hybrid (LDH) system prepared from 1,2-dipalmitoyl-sn-glycero-3-phosphocholine, PAMAM G4.0, and 1,2-dipalmitoyl-sn-glycero-3-phosphocholine (DPPC). Encapsulated PTX via the LDH system showed greater therapeutic efficacy in IGROV1 and SKOV3 ovarian cancer cell lines in vitro and an IGROV1 ovarian tumour xenograft model in vivo [44].

A high expression of carbonyl reductase 1 (CBR1) in ovarian cancer is associated with good prognosis, Kobayashi et al. (2015) employed a gene therapy with CBR1 DNA and PAMAM dendrimer. The proliferation of human epithelial ovarian carcinoma cell lines, HRA and DISS, was significantly reduced after treatment with the CBR1/PAMAM dendrimer. Moreover, inhibition of the dissemination and proliferation of malignant cells was observed in mice receiving dendrimer/DNA complexes [45].

In general, nanoparticles are more effective than free drugs as a therapeutic option. However, passive targeting, based solely on the EPR effect, has a number of limitations. To overcome these challenges, the engineering of nanoparticles with active targeting ligands is currently an important issue of research.

**Table 1:** Clinical trials using doxorubicin nanoformulations in ovarian cancer treatment.

Name	Compounds	Type of Ovarian Cancer	Phase	Clinical Trial Number
PLD and SB-485232	pegylated liposomal doxorubicin + interleukin 18	Epithelial ovarian cancer	1	NCT00659178
PLD and ATI-0918	pegylated liposomal doxorubicin + liposomal formulation of doxorubicin hydrochloride	Ovarian cancer that has progressed or recurred after platinum-based chemotherapy	1	NCT01715168
PLD and MK-4827	pegylated liposomal doxorubicin + niraparib	Platinum-resistant/refractory HGSOC	1	NCT01227941
PLD and Yondelis	pegylated liposomal doxorubicin + trabectedin	Platinum-sensitive advanced-relapsed epithelial ovarian cancer; advanced-relapsed ovarian cancer	3	NCT01846611 NCT00113607 NCT02394015
PLD and Avastin	pegylated liposomal doxorubicin + bevacizumab	Platinum-resistant/refractory ovarian cancer; platinum-sensitive ovarian cancer	1, 2	NCT00846612 NCT00945139 NCT02163720
PLD and Fludarabine	pegylated liposomal doxorubicin + fludarabine	Platinum-resistant or refractory ovarian cancer	2	NCT03335241
PLD and BAY94–9343	pegylated liposomal doxorubicin + anetumab ravtansine	Recurrent mesothelin-expressing platinum-resistant ovarian cancer	1	NCT02751918
PLD and Hycamtin	pegylated liposomal doxorubicin + topotecan hydrochloride	Recurrent epithelial ovarian carcinoma	3	NCT01840943
PLD and pazopanib	pegylated liposomal doxorubicin + pazopanib	Advanced relapsed platinum-sensitive or platinum-resistant ovarian cancer	1/2	NCT01035658
PLD and EC-145	pegylated liposomal doxorubicin + vintafolide (conjugate of desacetylvinblastine monohydrazide with folic acid	Platinum-resistant ovarian cancer	2, 3	NCT00722592 NCT01170650
PLD and Ashwagandha	pegylated liposomal doxorubicin + withaferin A	Recurrent ovarian cancer	1, 2	NCT05610735
PLD and Carboplatin	pegylated liposomal doxorubicin + carboplatin	Ovarian cancer recurrent within six to twelve months after initial carboplatin and paclitaxel chemotherapy	2	NCT00780039
PLD and Telcyta	pegylated liposomal doxorubicin + TLK286	Platinum refractory or resistant ovarian cancer	1/2	NCT00052065
PLD or Hycamtin vs. Telcyta	pegylated liposomal doxorubicin, topotecan hydrochloride, TLK286	Platinum refractory or resistant ovarian cancer	3	NCT00057720
PLD and IMC-3 G3	pegylated liposomal doxorubicin + olaratumab	Platinum refractory or resistant ovarian cancer	2	NCT00913835
PLD and BMS-247550	pegylated liposomal doxorubicin + ixabepilone	Advanced epithelial ovarian cancer, previously treated with platinum and a taxane	1/2	NCT00182767
PLD vs. AZD2281	pegylated liposomal doxorubicin, olaparib	*BRCA1/2*-positive advanced ovarian cancer patients who have failed previous platinum-based chemotherapy	2	NCT00628251
PLD and AZD2281	pegylated liposomal doxorubicin + olaparib	Platinum-resistant advanced ovarian cancer	2	NCT03161132 [46]
PLD and Vectibic	pegylated liposomal doxorubicin + panitumumab	Platinum-resistant epithelial ovarian cancer with *KRAS* wild-type	2	NCT00861120
PLD and bortezomib	pegylated liposomal doxorubicin + bortezomib	*BRCA* wild-type platinum-resistant recurrent ovarian cancer	2	NCT03509246 [47]
PLD and BIBF 1120	pegylated liposomal doxorubicin + nintedanib	Platinum-resistant ovarian cancer	1	NCT01485874

### 2.2. Therapeutic Strategies Based on Nanoparticles Modified with Active Targeting Ligands for Ovarian Cancer

To reduce the risk of significant off-target delivery, the identification of receptors specifically expressed in ovarian cancer tissues is necessary. Human epidermal growth factor 2 (HER2) or v-erb-b2 avian erythroblastic leukaemia viral oncogene homologue 2 (ERBB2), located on chromosome 17 q12–21 [48], is a transmembrane tyrosine kinase receptor protein belonging to the epidermal growth factor (EGF) receptor family [49]. HER2 is implicated in cell proliferation and survival, migration, adhesion, and apoptosis through activation of the Ras/mitogen-activated protein kinase (MAPK) and phosphoinositide-3-kinase (PI3 K)/AKT pathways, as well as protein kinase C (PKC) [50,51]. Overexpression of HER2 is commonly detected in multiple tumour types, in particular, breast [52], pancreatic [53], endometrial, and colorectal cancer. Notably, several different ovarian cancer types (including serous, mucinous, and clear-cell epithelial ovarian cancer) are characterised by HER2 overexpression [54], which is associated with higher invasion, chemoresistance, aggressiveness, and overall poor survival [54,55,56]. Tumours are classified as HER2-positive when more than 10% of cells exhibit an intensity greater than 3+ or 2+ with in situ hybridisation, signifying *ERBB2* gene amplification [57]. Clinical trials using targeted therapy with antibodies against HER2 have revealed poor responses in patients [58,59], which could be attributed to weaker overall HER2 expression in ovarian cancer than in HER2-positive breast cancer. On average, human ovarian cancer tissues contain 10−20% of HER2-positive tumour cells [58]. Consequently, current research has focused on the development of optimal strategies based on nanoparticle delivery systems.

Folate-binding proteins or the FA receptor (FR) family consists of four proteins: FRα (257 aa, 30 kDa, *FOLR1*), FRβ (255 aa, 29 kDa, *FOLR2*), FRγ (245 aa, 28 kDa, *FOLR3*), and FRδ (250 aa, 28.6 kDa, *FOLR4*). FRα and FRβ are equipped with glycosylphosphatidylinositol (GPI) anchors. FA receptors bind with high affinity to FA and its derivatives, such as 5-methyltetrahydrofolate (methylfolate, 5-MTHF) and folate-conjugated compounds [60]. FRα mediates cell growth, division, and proliferation [61] and displays low expression in normal human tissues [62]. This protein has been identified in the plasma membrane of epithelial cells, in particular, epithelia of the choroid plexus, proximal kidney tubules, fallopian tube, uterus and epididymis, acinar cells of the breast, submandibular salivary and bronchial glands, type I and II pneumocytes in the lung, trophoblasts in the placenta, germinal epithelium of the ovary, epithelium of the fallopian tube, surface epithelium of the uterus, surface epithelium of the endocervix, and glandular epithelium of the cervix [63]. Overexpression of FRα has been reported in several cancer types, including ovarian, endometrial, breast, and lung cancers [64,65,66]. Approximately 90% of ovarian carcinomas overexpress FRα [67].

CD44 is another cell surface glycoprotein, hyaluronate receptor, implicated in tumour stemness, recurrence, and drug resistance of ovarian and breast cancer. CD44 is involved in matrix-cell signalling, cell migration, and intercellular adhesion pathways. Overexpression of CD44 is associated with poor prognosis, while its depletion significantly suppresses tumour proliferation in ovarian cancer [68]. Hyaluronic acid (HA) is frequently added to the surface of NPs to target CD44, particularly in ovarian cancer and stem cells, to prevent metastasis and overcome drug resistance. HA, a linear polysaccharide composed of units of D-glucuronic acid and N-acetyl-D-glucosamine [69], is widely used for drug carrier modification owing to its bioavailability and biocompatibility. HA-based drug delivery systems can be obtained by covalent bond modification or electrostatic attraction [21].

Angiogenesis, the process of new blood vessel formation, plays a significant role in cancer development and spread. This mechanism involves interactions between a range of specific molecules (such as platelet-derived growth factor, fibroblast growth factor, and vascular endothelial growth factor) and their corresponding receptors (e.g., the Tie2 receptor). Research efforts to prevent tumour angiogenesis, a crucial mediator of disease progression, have primarily focused on the VEGF pathway [70,71]. The VEGF family is composed of seven proteins: VEGF-A–E and placental growth factors 1 and 2 (PIGF-1 and PIGF-2). These proteins exert their effects by activating signalling pathways through interactions with tyrosine kinase receptors on the surface of endothelial cells, known as vascular endothelial growth factor receptors (VEGFR) 1–3. Activation occurs when the ligand binds its receptor, triggering receptor dimerisation and subsequent signalling. In ovarian cancer, VEGF signalling involves PI3 kinases, MAPK, and components of the Janus kinase-signal transduction and transcription activation (JAK-STAT) pathway. In addition to VEGF/VEGFR complex proteins, src kinases, and phospholipase C contribute to signalling interactions. These proteins enhance vascular permeability and interact with extracellular signal-regulated kinase (Erk)/MAPK molecules [72].

Numerous growth factors other than VEGF, such as platelet-derived growth factor (PDGF), epidermal growth factor (EGF), fibroblast growth factor (FGF), hepatocyte growth factor (HGF), and angiopoietins, additionally contribute significantly to angiogenesis. These pathways should be taken into consideration when developing combination therapies to counteract resistance to anti-VEGF treatment [73].

#### 2.2.1. Human Epidermal Growth Factor Receptor 2

To enhance the internalisation of PLD, Lin et al. (2021) engineered a noncovalent form of the drug consisting of a humanised bispecific antibody, with one of the arms of Fab directed against methoxyl-polyethylene glycol (mPEG) and the other arm containing a single-chain fragment variable (scFv) against HER2 antigen. These modifications not only increased cytotoxicity against HER2-overexpressing SKOV3 ovarian cancer cells but also promoted the accumulation of the drug in a HER2-positive tumour xenograft mouse model [74].

Liposomal delivery of gene therapy for ovarian cancer is a technique known to replace or change the activity of genes. It has been shown to reduce tumour size, weight, and improve survivability. Significant progress has been made in the delivery of plasmid DNA, mRNA, small interfering RNA (siRNA), microRNA (miRNA), or antisense oligonucleotide therapies for cancer treatment. RNAi technologies are being developed to silence ovarian cancer oncogenes and mutated tumour suppressor genes by inactivating their complementary mRNA [75]. One of the drug delivery methods using DOX and siRNA is based on gold nanoparticles engineered with the recombinant bifunctional fusion protein TRAF(C) (TR). The thiol moiety at the C-terminal cysteine facilitated conjugation to the nanoparticle with concomitant loading of DOX and siRNA targeting *ERBB2*. Studies conducted in vivo on SKOV3 xenograft nude mice demonstrated target-specific uptake, low off-site toxicity, and significant tumour suppression [76].

Theranostic nanoparticles integrate diagnostic and therapeutic elements into a single compound. Imaging nanoparticles (gold nanocages, iron oxide nanoparticles, and quantum dots) can be conjugated to anticancer drugs. Another alternative is labelling therapeutic nanoparticles with imaging contrast agents, such as fluorescent dyes or radioisotopes [77]. Nanoparticles consisting of a HER2 affibody radiolabelled with a unique near-infrared dye (NIR-830), used as a PET imaging probe conjugated to magnetic iron oxide nanoparticles, have been tested in HER2-positive SKOV3 and HER2-negative OVCAR3 tumour xenograft models. Affibody molecules are small (6.5–7 kDa) affinity proteins that utilise a cysteine-free three-helical scaffold [78,79]. The Np induced an increase in tumour site accumulation through active targeting of tumour cell receptors [80]. Subsequently, the activity of this nanoparticle was enhanced by cisplatin. The authors reported an increase in drug delivery to both primary ovarian tumours and distant and peritoneal metastases, as well as 88% inhibition of tumour growth at a dose of 5 mg/kg [81].

Trastuzumab, a recombinant humanised monoclonal antibody, impedes HER2 activity by inhibiting cleavage of the extracellular domain. This molecule was initially established as a standard therapy for HER2-positive breast cancer [82]. HER2-targeted PGLA polymeric nanoparticles loaded with cisplatin appear to exert greater cytotoxicity against SKOV3 cells than against HER2-negative breast cancer HCC70 cells [25].

#### 2.2.2. Folic Acid Receptors

Accordingly, in addition to its potential utility as a clinical biomarker of ovarian cancer, growing interest has been focused on FRα as a target for therapeutic interventions. Several antibodies that specifically bind FRα, such as mirvetuximab, soravtansine, and farletuzumab, have been evaluated in clinical trials [83,84].

Other research of interest concerns the potential applications of NPs loaded with polyphenols. The polyphenol epigallocatechin gallate (EGCG) is the main component of green tea. Chuan et al. (2021) modified EGCG with folic acid for the preparation of FA-PEG-EGCG (FPE)/doxorubicin nanoparticles. Data from both in vitro and in vivo experiments showed enhanced toxicity in ovarian cancer cells and xenografts compared to nanoparticles with no folic acid modification [85].

Nanoceria (NCe), nanoparticles of cerium oxide represent another type of nanoparticle developed for the treatment of ovarian cancer. These metal oxide-based nanoparticles of cerium oxide possess outstanding antioxidant properties due to an “oxidation switch” on their surface, which promotes an automatic shift between Ce^3+^ and Ce^4+^ oxidation states [86]. NCe particles conjugated with folic acid alone and in combination with cisplatin have been developed. Folic acid conjugation of NCe (NCe-FA) led to an increase in internalisation and a decrease in viability of the ovarian cancer cell lines A2780, SKOV3, OVCAR3, and C200. Moreover, a combination of NCe with cisplatin was effective in reducing proliferation and angiogenesis in a xenograft mouse model [87].

Multiple studies have focused on the conjugation of PEG with PLGA. As stated above, this step has numerous benefits, including aqueous solubility, stability in opsonisation, immunogenicity, and enhancement of half-life. Accordingly, PLGA-PEG nanoparticles were further conjugated with FA to specifically bind folate receptors and were loaded with the natural isoflavone genistein found in soybean products. PLGA-PEG-FA clearly induced an increase in cellular uptake and cytotoxicity in SKOV3 cells [88].

An innovative method for nanoparticle formation was presented by Ak et al. [89]. The group synthesised DOX-loaded and glucose/gluconic acid-coated magnetic Fe_3_O_4_ nanoparticles, which were coated with erythrocyte membrane vesicles and folate ligands anchored to the surface for targeting receptors. The results are promising because the delivery system showed a controlled drug release profile and greater cytotoxicity against A2780 and OVCAR3 cells than non-targeted nanoparticles. Furthermore, this delivery strategy was more effective in xenograft nude mice, resulting in greater tumour reduction relative to free doxorubicin [89].

Nanoparticles could be utilised to not only treat but also to accurately detect the origin of cancer. Magnetic resonance imaging (MRI) provides three-dimensional resolution of tissues, facilitating the detection and differentiation of pathological changes from healthy tissues. Contrast agents are categorised into two main groups based on longitudinal (T1) and transverse (T2) relaxation processes. T1 contrast agents are characterised by shortened T1 relaxation time with bright images and T2 agents by a shortened T2 relaxation time with dark images, otherwise known as ‘negative contrast agents’ [90,91]. Superparamagnetic materials, such as iron oxide, can be used as T2 contrast agents owing to their superior biocompatibility and greater sensitivity at lower concentrations relative to other molecules, such as gadolinium complexes [92]. FA-targeted iron oxide (Fe_3_O_4_) nanoparticles have been developed as T2 negative contrast agents for in vivo MRI with the aim of accurately detecting ovarian tumour tissues in a xenograft tumour model. These nanoparticles have low toxicity and biodegradable properties, and may be used as a diagnostic system for early and specific detection and treatment of ovarian carcinomas [93]. A unique nanoparticle targeting the FA receptor is currently in clinical trials. ELU001 is described as a C’Dot Drug Conjugate (CDC) and consists of ~13 folic acid targeting moieties and a payload of ~22 molecules of the topoisomerase-1 inhibitor exatecan. Folic acid and exatecan are covalently bound by non-cleavable and cathepsin-B cleavable linkers, respectively, to short polyethylene glycol chains that surround the C’Dot’s silica core. ELU001′s high avidity is designed to promote binding to FRα on the surface of FRα overexpressing cancer cells with a wide range of antigen expression, including high, moderate and low expressing tumour cells. The first-in-human trial, ELU-FRα-1, is currently under clincal trail in studies on, among others, ovarian cancer patients overexpressing FRα and topoisomerase 1 inhibitor-sensitive, NCT05001282 [94].

Another tumour-targeting therapeutic agent discovered for ovarian cancer treatment is F-LP/pMP. The new nanoparticle was based on the human telomerase reverse transcriptase (hTERT) promoter, which could preferentially direct target gene expression in human ovarian cancer cells that possess high levels of telomerase. The second component was the matrix protein (MP) from the vesicular stomatitis virus (VSV) which exerts an antitumour effect by inducing tumour cell apoptosis. A tumour-targeting gene delivery system was constructed upon FRα -targeted lipoplexes that includes a vector with hTERT to encode a gene (pMP). F-LP/pMP led to the induction of tumour cell apoptosis, inhibition of tumour cell proliferation, and suppression of angiogenesis in SKOV-3 cells [95]. It also used oligonucleotide microRNA mimics (OMMs) to increase the levels of downregulated miRNAs. Targeting miR-18 a with OMM-loaded folate-liposomes has therapeutic potential in women diagnosed with cisplatin-resistant ovarian cancer. Intraperitoneal injections of miR-18 a-OMM-loaded folate-conjugated liposomes significantly reduced the tumour weight and the number of nodules in ovarian cancer-bearing mice when compared with a control-OMM group [96]. FR-targeted liposome (F-LP) in another study was also used to deliver CRISPR plasmid DNA co-expressing Cas9 and single-guide RNA targeting the ovarian cancer-related DNA methyltransferase 1 gene (*gDNMT1*). F-LP bound the *gDNMT1* plasmid and formed a stable complex (F-LP/*gDNMT1*). F-LP/*gDNMT1* effectively mutated endogenous DNMT1 in vitro and then expressed Cas9 endonuclease and downregulated DNMT1 in vivo in paclitaxel-sensitive and resistant ovarian cancers [97].

#### 2.2.3. CD44, a Homing Cell Adhesion Molecule

In previous studies, lipids, liposomes, and albumin NPs coated with HA showed enhanced efficacy of transferred compounds and endocytotic absorption that effectively suppressed ovarian tumour growth, both in vivo and in vitro [98]. The effects of the surface architecture of the modified NPs on their binding to ovarian cancer cells overexpressing CD44 were investigated in a separate study. These molecules were constructed from anionic liposomes with 5 kDa HA by covalent conjugation via surface coupling or electrostatic self-assembly using the layer-by-layer (LbL) adsorption method. Enhancement of NP-cell associations was observed with the self-assembly LbL technique, particularly for higher molecular weight (≥10 kDa) HA. The formulation generated using 100% bottlebrush polymer (no PEG side chains) showed the highest binding avidity [99].

Efforts to construct nanoparticles that specifically target CD44 are currently in progress. In the fluorescently labelled serum albumin-HA conjugate, HA serves as both the active targeting component and hydrophilic external domain in the amphiphilic nanocarrier. Serum albumin is the major natural nanovehicle of various compounds in mammalian blood. HA-serum albumin conjugates containing a diagnostic tracer (FITC) have been developed. In a study by Edelman et al. (2019), these NPs were loaded with PTX and used to treat the ovarian adenocarcinoma cell lines SKOV3 and A2780. Receptor-mediated endocytosis of FITC-HA-bovine serum albumin (BSA) conjugates into human ovarian cancer cells was achieved through the CD44 receptor [100]. Another novel approach is the application of polylactic acid (PLA), one of the FDA-approved polymers commonly used for drug delivery systems and prodrug engineering. PLA was conjugated with PTX via the esterification process to generate a PLA-PTX prodrug by Sun et al. (2022). NPs encapsulating PLA-PTX were prepared using a one-step nanoprecipitation technique. HA-decorated matrix (HA-PEG-PLA) was additionally introduced for the generation of a CD44-specific delivery system (HA-PLA-PTX NP), and the delivery efficacy was assessed using CD44-negative A2780 and CD44-positive SKOV3 cell lines. The PLA fragment was effectively used to overcome the poor water solubility. Notably, large-scale surface PEGylation inhibited identification by the mononuclear phagocyte system (MPS) and hydrophobic and electrostatic interactions with plasma proteins, thus prolonging the shelf life of encapsulated PTX-PLA prodrugs in the blood circulation. Although these nanoparticles were less potent than Taxol in vitro due to the additional step of releasing active PTX, they demonstrated superior anticancer efficacy in vivo [68].

Nanoparticles specifically targeting ovarian cancer have been engineered to solve the problem of chemotherapy resistance. Zhao et al. (2019) investigated the application of hyaluronic acid-coated PEI nanoparticles for curcumin and PTX co-delivery to reverse drug resistance. PTX and curcumin loaded into PEI-stearic acid (SA)HA micelles were prepared using probe-type ultrasonication. In this system, hyaluronic acid could target cancer cells containing membrane-bound CD44 receptors as an active ligand, while the amphiphilic graft copolymer PEI-SA, containing a hydrophobic segment of SA and hydrophilic block of PEI, self-assembled into nanoparticles. Biocompatible stearic acid, an endogenous long-chain saturated fatty acid, and 16-modified polyethyleneimine served as vehicles for the delivery of PTX and curcumin. The drugs exerted synergistic anticancer effects in ovarian cancer cells (SKOV3) and multidrug-resistant variant cells (SKOV3-TR30) in vitro and effectively induced antitumour responses in nude mice bearing ovarian tumours in vivo. These NPs could reverse resistance to PTX to a significant extent by suppressing overexpression of P-gp [101]. HA-conjugated polyethyleneimine (PEI)/PEG nanoparticles were additionally designed for in vitro delivery of MDR1 siRNA and PTX to chemoresistant OVCAR8 TR cells. HA-PEI/HA-PEG nanoparticles loaded with siMDR1 efficiently targeted CD44 receptors on drug-resistant ovarian cancer cells, causing efficient suppression of MDR1 and inhibition of P-gp activity. Consequently, tumour cells were more sensitive to PTX-mediated inhibition in a mouse model of MDR ovarian cancer [102].

Shah et al. (2013) developed a polypropylene imine (PPI) dendrimer-based drug delivery system using PTX conjugated to PPI (PTX-PPI) and CD44-targeted siRNA-PPI complexes (CD44 siRNA-PPI). This siRNA suppressed both CD44 and MDR1 mRNA expression, which significantly improved the ability of PTX to induce apoptosis while preventing adverse effects in healthy organs. After a 28-day period of exposure to monotherapy, PTX-PPI, or CD44 siRNA-PPI, tumours derived from cells isolated from the malignant ascites of patients with advanced ovarian carcinoma continued to grow. The combination of PTX and CD44 siRNA delivered by the nanocarrier led to almost complete tumour shrinkage [103]. Next, dual-targeted and glutathione (GSH)-responsive nanoparticles (SSBPEI–DOX@siRNA/iRGD–PEG–HA) were developed with the aim of efficient and specific delivery of doxorubicin and small interfering RNA cocktails (siRNAs for survivin, Bcl-2, and ABCG2) to ovarian cancer stem cells (CSCs), both in vitro and in vivo. A2780/cisplatin-resistant ovarian cancer cells cultured under serum-free conditions that formed stem cell microspheres were subcutaneously injected into BALB/cJ nude mice. NPs were generated by the electrostatic assembly of anionic siRNAs and cationic disulfide bond crosslinking-branched polyethyleneimine-doxorubicin (SSBPEI–DOX) as a core. The disulfide bond (–SS–) in SSBPEI–DOX could be specifically reduced to promote the controlled release of siRNA and DOX caused by glutathione reductase in the tumour microenvironment. IRGD peptides and hyaluronic acid (HA) mediate CSC targeting via specific binding to neuropilin-1 (NRP1) and CD44 to enhance delivery [104]. The proangiogenic mediator NRP-1 is potentially linked to the development of metastatic cancer, and its upregulation in ovarian cancer tissue is correlated with poor prognosis [105]. Cell surface-associated proteases cleave iRGD polypeptides containing the CendR motif (CRGDK) that binds NRP1. In this way, SSBPEI–DOX@siRNA/iRGD–PEG–HA could significantly reduce metastasis and invasion, leading to improved tumour regression [104].

A similar system is based on short hairpin RNAs (shRNAs). Focal adhesion kinase (FAK) and CD44 are common targets for shRNA-mediated knockdown therapy. FAK, a 125 kDa nonreceptor protein tyrosine kinase, is an important mediator of growth factor signalling, proliferation, migration, invasion, and survival of tumour cells. PLGA nanoparticles (PLGANPs) deliver therapeutic agents directly into the cytoplasm and nucleus of tumour cells, avoiding the endolysosomal pathway. In a study by Zou et al. (2013), PLGANPs were conjugated with shRNA for efficient expression and knockdown of genes in ovarian SKOV3 cells using a recombinant plasmid vector. The authors demonstrated that silencing FAK and CD44 with shRNA led to significant inhibition of the growth of human ovarian cancer xenografts in nude mice without systemic toxicity, highlighting the safety of PLGANPs. Data from Ki-67 staining and TUNEL assays consistently showed that silencing FAK and CD44 led to a significant decrease in cell proliferation and an increase in apoptosis [106]. Blocking of the CD44 gene via RNA interference (RNAi) was reported to significantly inhibit ovarian cancer cell growth and tumour blood vessel formation and reduce disease recurrence and metastasis [107]. Cisplatin/CD44-shRNA nanoliposomes were further prepared using PEG-modified manganese zinc ferrite nanoparticles (PEG-MZF-NPs). Tumour hyperthermia was achieved through the use of magnetic nanoparticles under the action of an external magnetic field. For the treatment of ovarian cancer, HO8910 cells were simultaneously administered shRNA and cisplatin using magnetic fluid hyperthermia (MFH). These NPs take advantage of magnetic response and temperature control, CD44-shRNA gene therapy, cisplatin chemotherapy, and magnetic thermotherapy. The results showed that VEGF, survivin, BCL-2, and BCL-xl proteins were significantly decreased after PEG-MZF-NP treatment, while caspase-3 and caspase-9 protein levels were markedly increased, both in vitro and in vivo, which resulted in ovarian tumour growth inhibition, restrained proliferation, and invasion, and promotion of cell apoptosis [108].

#### 2.2.4. Vascular Endothelial Growth Factor

A promising strategy less likely to cause resistance in endothelial ovarian cancer cells involves indirect disruption of tumours by compromising their vasculature [109]. An example of a targeted agent for the tumour vasculature is bevacizumab (BVC), a monoclonal antibody specific to VEGF-A. An FDA-approved drug for treating colon, lung, kidney, and brain cancers, bevacizumab, clearly illustrates the success of targeting the blood vessels of tumours. However, anti-VEGF therapy has not yielded the anticipated efficacy, with resistance observed in both tumour cells and elements of the tumour microenvironment [110].

To demonstrate the efficacy of BVC against ovarian cancer, researchers developed innovative mesoporous silica nanoparticles (MSN) coupled to tumour endothelial marker 1 (TEM1)/endosialin (Ab-/scFv). The resulting MSN possessed nanoscale dimensions and a spherical structure and exhibited controlled drug release properties at pH 7.4 [111]. Moreover, BVC-MSN/Ab enhanced the uptake of BVC by OVCAR-5 cancer cells, leading to a more widespread cellular distribution of the drug. Notably, the Ab-conjugated MSN exhibited superior anticancer effects, inducing significant apoptosis in a time- and concentration-dependent manner. Additionally, colony formation was markedly inhibited by BVC-MSN/Ab. Importantly, the use of BVC-MSN/Ab led to a marked increase in the proportion of cells in the G2/M phase of the cell cycle, indicating a promising anticancer efficacy profile [111].

NCe molecules, nanoparticles composed of cerium oxide characterised by their antioxidant properties, are under consideration for therapeutic application in ovarian cancer. In A2780 ovarian cancer cells, NCe significantly suppressed the generation of reactive oxygen species (ROS) and mitigated the migration and invasion of SKOV3 cells mediated by growth factors (SDF1, HB-EGF, VEGF165, and HGF) while having no effect on cell proliferation. Additionally, NCe treatment hindered VEGF165-induced proliferation, capillary tube formation, activation of VEGFR2, and matrix metalloproteinase-2 (MMP2) in human umbilical vascular endothelial cells (HUVEC). In nude mice injected intraperitoneally with A2780 cells, treatment with NCe (0.1 mg/kg body weight) led to a notable reduction in tumour growth, accompanied by a decrease in tumour cell proliferation, as evident from smaller tumour sizes and reduced Ki67 (proliferation marker) staining. The decrease in tumour mass was linked with a reduction in angiogenesis, observed as diminished CD31 staining and specific apoptosis of vascular endothelial cells [112]. In an ovarian cancer model, NCe particles administered intraperitoneally suppressed angiogenesis, even at a low dose of 0.1 mg/kg. While the application of NCe inhibited VEGF-induced proliferation, capillary tube formation, and activation of MMP2, the precise therapeutic mechanism of action remains unclear. NCe may influence VEGF-mediated signalling events that are sensitive to redox changes. Importantly, high doses of NCe up to 250 mg/kg have been administered without causing noticeable toxicity, suggesting that these nanoparticles could serve as viable substitutes for small-molecule antiangiogenic agents that pose toxicity risks [113].

An aptamer designated Macugen binds to VEGF, preventing interactions with its receptors, VEGFR-1 and VEGFR-2. The modification of magnetic nanocrystals with this aptamer facilitates the precise identification of angiogenic vasculature using MRI (Hu et al., 2022). A hydrogel containing thiolated HA-PEG diacrylate with immobilised RGD peptides for cell adhesion and anti-VEGF-R2 DNA aptamers has also been developed [114].

Research focusing on ovarian cancer has unveiled a pioneering aptamer-siRNA chimera delivery system involving cationic Au-Fe_3_O_4_ nanoparticles [115]. This chimera, comprising a VEGF-RNA aptamer and Notch3 siRNA, binds to Au-Fe_3_O_4_ nanoparticles through electrostatic interactions. The resulting complexes exhibit enhanced Notch3 gene silencing efficacy, resulting in a greater antitumour effect. Furthermore, proficient delivery of Au-Fe_3_O_4_ nanoparticles demonstrates promise in overcoming resistance to cisplatin, highlighting their potential as targeted tumour therapy to combat multidrug resistance.

Dual-surfaced dumbbell-like gold magnetic (Au-Fe_3_O_4_) nanoparticles have been synthesised that function as intelligent photo-controlled drug carriers for precise aptamer delivery [116]. Through electrostatic absorption, DNA aptamers targeting VEGF and Au-Fe_3_O_4_ NP were assembled, enabling Apt-Au-Fe_3_O_4_ NP to specifically bind to SKOV-3 ovarian cancer cells. Following exposure to plasma resonance light (605 nm), aptamers were significantly released within cells, effectively enhancing the inhibition of tumour cell proliferation in vitro. These collective findings indicate that Apt-Au-Fe_3_O_4_ NPs hold considerable promise as targeted cancer hyperthermia carriers that can be remotely controlled, offering high spatial/temporal resolution [117].

Likewise, promising outcomes have been reported for liposomal nanocarriers incorporating siRNA against VEGF and kinesin spindle protein (ALN-VSP) in an earlier phase II trial. High tumour targeting was demonstrated based on the knockdown of protein expression, low toxicity profiles, and regression of liver metastases in ovarian cancer patients [113].

A study by Bhattacharya et al. (2022) aimed to enhance cellular uptake, targeting efficiency, and cytotoxicity by anchoring EGFR vIII (an anti-VEGF molecule) onto nanoparticles loaded with gemcitabine (GTB). Various cationic intravesical polymeric nanoparticles have been developed and examined for effective GTB delivery to treat ovarian cancer. Among these, chitosan and polysarcosine-fabricated nanoparticles (CS-PSar-NPs) displayed excellent drug loading and release profiles, coupled with effective cell internalisation and anticancer effects. Polymeric nanoparticles containing a combination of chitosan and polysarcosine have demonstrated promise for bioadhesive drug delivery. Furthermore, epidermal growth factor receptor variant III (EGFR vIII) was incorporated into these polymeric nanoparticles with the aim of achieving enhanced target specificity. Notably, among all nanoparticles with molecular modifications, CS-PSar-NPs-EGFRvIII displayed increased cell internalisation and accumulation in OVCAR-8 cells. These EGFR vIII-modified nanoparticles could potentially mitigate the toxicity of molecularly targeted GTB, offering a promising strategy for ovarian cancer treatment [118].

The challenging outlook for patients with drug-resistant ovarian cancer and the absence of targeted therapies have highlighted the urgency for alternative treatment approaches. Researchers have examined the potential repurposing of albendazole (ABZ), recognised for its anti-parasitic properties that disrupt microtubule formation, as an antiangiogenic drug (with activity as a VEGFR-2 inhibitor) for the treatment of ovarian cancer with ascites. However, the limited solubility of ABZ in water restricts its application in cancer therapy. To overcome this limitation, ABZ was combined with BSA, generating particles 7–10 nm (BSA-ABZ) and 200–250 nm (Nab-ABZ) in size. In vitro cell proliferation tests revealed that BSA-ABZ 10 nm was most effective in killing ovarian cancer cells (OVCAR3 and SKOV3), while exerting minimal negative effects on healthy ovarian epithelial cells (HOSE). Confocal microscopy and fluorescence-activated cell sorting (FACS) analyses further indicated enhanced uptake of BSA-ABZ (10 nm) by cancer cells [119].

## 3. PARP Inhibitor Nanoformulations

### 3.1. History and Mechanisms of Action

Poly (ADP-ribose) polymerase (PARP) enzymes are a family of proteins with high nuclear expression in mammalian cells [120]. The role of PARP in mediating DNA repair is crucial for the survival of cancer cells with HR deficiency due to *BRCA1/2* mutations, supporting its potential as an ideal target for therapeutic intervention [9]. In 2014, olaparib was the first FDA- and EMA-approved PARPi as a monotherapy for advanced germline *BRCA*-mutated ovarian cancer [121]. In 2017, its approval was expanded to maintenance therapy for reoccurring ovarian, fallopian, and primary peritoneal tumours regardless of *BRCA* status [122,123]. Olaparib gained approval for the treatment of germline *BRCA1/2* mutated HER2-negative breast and metastatic pancreatic cancer in 2018 and 2019, respectively [124,125]. Recently, olaparib received authorisation for HRD-positive metastatic castration-resistant prostate cancer [126]. Rucaparib, niraparib, and talazoparib have also been accepted for various clinical applications.

Rucaparib received accelerated approval in 2016 for the treatment of *BRCA1/2*-mutated advanced ovarian carcinoma and later as maintenance therapy in 2018 [127,128]. In 2020, this drug gained FDA approval for *BRCA1/2*-mutated metastatic castration-resistant prostate cancer [129]. Niraparib was accepted in 2017 for maintenance and expanded to late-line treatment in 2019 and further in 2020 for specific biomarker-independent cases [130]. Talazoparib represents a next-generation PARPi with approximately 100-fold higher lethality than first-generation drugs. Preclinical studies have demonstrated that talazoparib induces DNA damage in ovarian and colon cancers irrespective of *BRCA* mutations, thus expanding the therapeutic window of talazoparib in relation to other PARPi compounds. Despite gaining approval for treating metastatic germline *BRCA1/2* breast cancer in 2018, widespread acceptance for other malignancies has faced challenges due to the pronounced side effects of the drug [120]. Veliparib is currently in clinical trials, while fluzoparib, initially identified in 2017, began trials in 2019 for various solid tumours [130].

**Figure 5:**
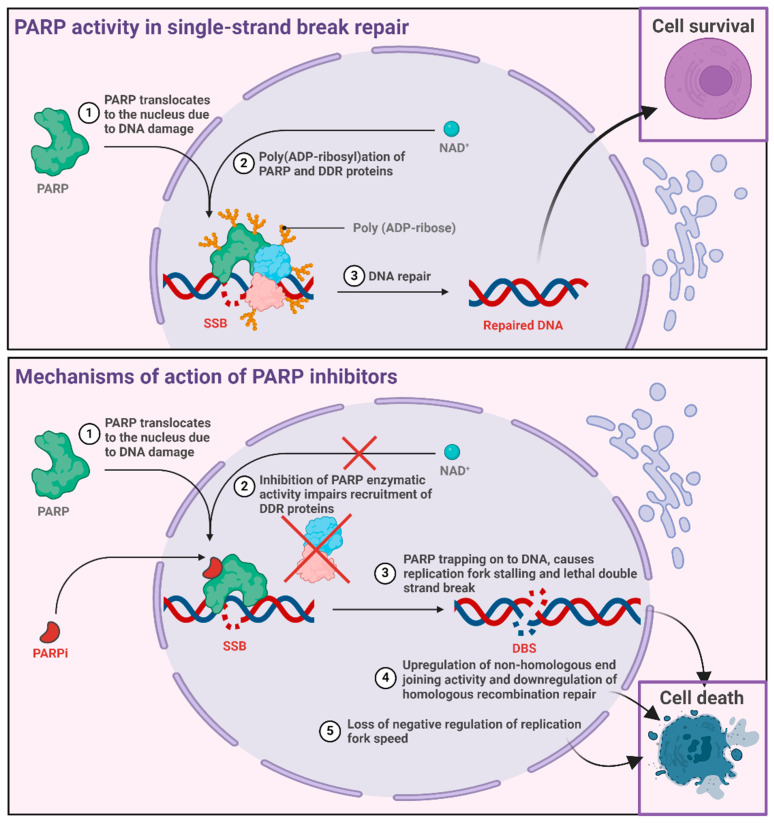
Proposed mechanism of action of PARP inhibitors.

PARPi compounds exhibit anticancer activity; however, the underlying mechanisms are complex and incompletely understood. Initially, it was proposed that inhibiting SSBs repair could induce lethality. However, conflicting evidence supports alternative pathways [130,131]. Replication fork stalling and PARP trapping have emerged as prominent theories, implicating the disruption of replication forks and competitive binding of PARPi to the NAD+ binding domain of PARPI. This trapping mechanism, linked to DNA lesions, contributes to the formation of DSBs and stalled replication forks in HR-deficient tumours (Figure 5) [132,133].

Inconsistent results have been reported from studies on the correlation between PARP trapping and cell line toxicity, casting doubt on its sole role in anticancer activity. Activation of the non-homologous end joining (NHEJ) repair pathway, specifically involving upregulation of NHEJ activity, is proposed as a synthetically lethal interaction in HR-deficient tumours. However, the effects of the simultaneous administration of a DNA-dependent protein kinase inhibitor and PARPi suggest a more complex relationship [130]. The disrupted processing of Okazaki fragments and replication fork speed offer another mechanism, in which the inhibition of replication fork regulators induces PARP1 accumulation and increases replication speed, potentially leading to cytotoxic single-stranded DNA gaps. Additionally, the disruption of PARP1 in transcriptional regulation adds complexity, affecting oncogenes implicated in cancer cell survival [130,134,135].

### 3.2. Encapsulated PARP Inhibitors for Ovarian Cancer Treatment

Liposomal preparations of PARP inhibitors have been extensively investigated as a treatment option for metastatic ovarian cancer. The estimated size of the sustained release formulation, NanoOlaparib, is 72.8 ± 5.8 nm. A study by Baldwin et al. (2018) showed high cytotoxicity of nanoOlaparib against cancer cells (404, a murine cell line) compared to free olaparib after direct administration into the peritoneal cavity, with accumulation in cancer tissues for up to 72 h after a single dose and 100% drug release over 8 days. Following IP delivery of NanoOlaparib to mice, the duration of response of animals to the PARP inhibitor increased from 2 weeks (upon administration as an oral formulation) to 3 weeks. Olaparib could not be obtained at the tumour site without overdosing owing to the sustained release profile of the nanoparticles [136]. Polymeric NPs represent a promising strategy for delivering PARPis. These nanoparticles can modulate drug release and enhance the targeting of cancer cells by altering tumour environment factors, such as pH and enzymatic activity. Moreover, polymer-based nanomaterials are highly stable, biodegradable, and biocompatible. The composition of PLGA-NPs can be adjusted to temporally control the release of cargo [137]. The photosensitizer, methylene blue (MB), and veliparib were co-encapsulated in PLGA nanoparticles to generate VMB-NPs. Photodynamic therapy (PDT) should eradicate tumour tissue within a few sessions to eliminate the risk of resistance. The hydrodynamic diameter of the polymeric NPs was determined to be 90 nm. VMB-NPs did not induce cytotoxicity in the dark. However, the viability of B16 F10-Nex2 cells was decreased by 36% upon irradiation (102 J/cm^2^, 660 nm) and treatment with VMB-NPs containing 1.0 µM MB and 8.3 µM veliparib [138]. Various self-assembled nanostructures have been exploited to achieve efficient delivery of PARP inhibitors. In this case, BSA was reacted with Ga^3+^ to form a complex. Gallic acid was added to the BSA-Ga^3+^ complex to generate the nanoformulation, followed by the linking of Olaparib through the hydrophobic effect of BSA. Previous studies have demonstrated that the self-assembled olaparib-Ga nanodrug suppressed the expression of ribonucleotide reductase M2 (RRM2), a gene associated with resistance, regulated cell death, and tumour immunity [139]. Furthermore, olaparib-Ga has been reported to activate the Fe^2+^/ROS/MAPK pathway and haeme oxygenase 1 (HMOX1) signalling, inhibit the PI3 K/AKT pathway, and enhance the expression of cleaved caspase-3 and BAX protein [140]. PARP inhibitors can also be delivered to OC cells in the form of metal-organic frameworks (MOFs). MOFs represent a class of hybrid multidimensional materials derived from metal ions or clusters connected by organic ligands [141,142]. These NPs consist of the high-Z element Hafnium (Hf) and the 1,4-dicarboxybenzene ligand (Hf-BDC), which are loaded with two DNA damage response (DDR) pathway inhibitors, talazoparib and buparlisib (TB@Hf-BDC-PEG). The hafnium ion (Hf^4+^) is a promising high-Z metal that readily chelates with certain ligands, such as photosensitisers [141]. TB@Hf-BDC-PEG augmented radiotherapy by increasing ROS production, which impeded the subsequent repair of DNA damage. Synergistic enhancement was demonstrated in vivo, whereby concurrent radiation with IV administration of TB@Hf-BDC-PEG improved tumour control and increased apoptosis. No toxicity was induced by the NPs [143].

### 3.3. Targeted Therapy by Encapsulated PARP Inhibitors

FR-targeted liposomes containing a combination of niraparib and doxorubicin were tested in a panel of ovarian cancer cell lines, including OVCAR8 (FRα-overexpressing) and HEYA8 (FRα non-overexpressing). Notably, the cellular uptake of folate-conjugated liposomes was greater than that of unconjugated control liposomes. However, this effect was not observed in the control HEYA8 cell line. Interestingly, the authors showed that the doxorubicin-niraparib combination induced antagonism in an HR-proficient cell line. Specifically, in the PEO4 cell line with a reverse mutation of *BRCA2*, the drugs exerted antagonistic effects at all tested concentration ratios [144].

Plectin is highly expressed and localised on the cell surface in advanced ovarian cancer. Plectin-targeted peptide (PTP)-conjugated nanoliposomes encapsulating the PARPi AZ7379 were prepared with sizes ranging from 110 to 120 nm and a zeta potential of 31 ± 1.6 mV. The peptides were conjugated to DSPE-PEG3400-maleimide. In mice bearing advanced subcutaneous and IP ovarian tumours (SKOV3 and OVCAR8), PTP liposomes incorporating AZ739 increased PARP inhibition, leading to slower tumour growth and an overall 3- and 1.7-fold decrease in tumour volumes [145].

A very interesting solution was recently proposed by Mensah et al. (2019), involving the use of the LbL NP platform for combined drug administration. In these experiments, PARPi BMN 673 (talazoparib) was combined with cisplatin and encapsulated in novel LbL polymeric liposomal nanoparticles, formulated with a lipid self-assembly core based on 1,2-distearoyl-*sn*-glycero-3-phosphocholine (DSPC), 1-palmitoyl-2-oleoyl-*sn*-glycero-3-phospho-(1′-rac-glycerol) sodium salt (POPG), and cholesterol, and subsequently layered with the polycation PLL. Additionally, the nanoparticles were coated with HA to increase the affinity of vesicles for CD44 receptors overexpressed in ovarian cancer. These nanoparticles mainly targeted HGSOC tumours, which are commonly resistant to typical platinum-based therapy. In vitro experiments were conducted using A431, COV362, COV318, JHOS2, and Kuramochi ovarian cell lines, and in vivo studies were performed on female nude mice with CD44-expressing OVCAR8 orthotopic xenografts. Both talazoparib and cisplatin were localised within the liposomal core and bilayer. Moreover, PLL improved the release of the drug at the tumour pH, which was lower than that in normal tissues. This type of formulation clearly improved the therapeutic efficacy of nanoparticles against ovarian cancer in mice. The drugs encapsulated in LbL NPs exhibited an extended half-life in blood circulation, gradual release, and targeted simultaneous selective delivery to HGSOC cells. Compared to free drugs, LbL NPs induced a significant reduction in metastasis, increased survival in mice, and lowered systemic toxicity, indicating a strong synergistic effect of this drug delivery system. Nanoparticles first released PARPi and then cisplatin, resulting in the complete blockade of DNA repair by HR and the effective induction of apoptosis, and consequently, synthetic lethality. Such an effect cannot be achieved with conventional methods of administering these anticancer drugs, which differ in characteristics since PARPi is a hydrophobic agent, while cisplatin is a hydrophilic drug [146].

## 4. Co-Encapsulation of Drugs as Combined Therapies for Ovarian Cancer

The effective use of PARPi in clinical practice is limited by the acquisition of development resistance due to the presence of compensatory pathways. One strategy to overcome this difficulty is to use combination therapy with other drugs that act synergistically against ovarian cancer [147]. Combination chemotherapy has been actively employed in the management of cancer for more than 50 years. In order to achieve optimal efficacy, drugs with different mechanisms of action are integrated. For example, cisplatin (a key platinum agent for ovarian cancer therapy) is often used in conjunction with PARPis to effectively reduce the development of multidrug resistance. However, the combination of PARPi and chemotherapeutics in the free form has some drawbacks. A number of studies have confirmed that although the combination of PARPi (AZD2281 and BMN 673) with cisplatin is effective, several obstacles prevent the full exploitation of the synergistic effects [148]. For example, the two drugs have different hydrophobic-hydrophilic properties, resulting in distinct biodistribution profiles when administered in the free form. PARPis are highly hydrophobic, with limited bioavailability and a relatively rapid plasma clearance rate. In contrast, cisplatin has hydrophilic properties, leading to different tumour delivery times and concentrations of the delivered drugs, which can significantly affect the efficacy of treatment. The distribution of drug combinations using nanocarriers can significantly increase the potency of treatment while reducing systemic toxicity [149].

Another study by Novohradsky et al. (2018) demonstrated extremely high efficacy of the PARPi, olaparib, encapsulated together with the antitumour platinum drug, carboplatin, in PEGylated liposomes, a formulation termed OLICARB. Experiments were conducted on two ovarian cancer cell lines, specifically, A2780 (cisplatin-sensitive) and A2780 cis (acquired resistance to cisplatin), with different molar ratios of olaparib:carboplatin (OLICARB (1:1) and OLICARB (2:1)). The results showed that olaparib and carboplatin encapsulated in PEGylated liposomes had significantly higher toxicity than free drug combinations, irrespective of the molar ratio of the drugs (3–13 fold). The most significant differences were observed for tumour cells resistant to cisplatin (A2780 cis). Significantly greater synergism was additionally confirmed (by analysing the CI coefficient) in the case of OLICARB compared to the use of free drugs or drugs encapsulated individually in the same type of liposomes. Additionally, OLICARB nanoparticles displayed marked selectivity towards tumour cells relative to normal cells. The antitumour effect of both drugs used in combination with OLICARB nanoparticles was correlated with DNA damage. The potentiation of carboplatin toxicity by olaparib in cancer cells was a consequence of the accumulation of cytotoxic changes in DNA due to the inhibition of carboplatin-damaged DNA repair processes by olaparib [150].

Another drug nanocarrier for the combined delivery of niraparib (NIRB) and cisplatin (Cis) is a Mn-based MOF (Mn-MOF) with transferrin (Tf) conjugated to the surface (Tf-Mn-MOF@Nira@CDDP; MNCT). MNCT effectively targets the tumour site, consumes glutathione (GSH), which is highly expressed in drug-resistant cells, and subsequently decomposes to release the encapsulated niraparib and cisplatin. These drugs synergistically promote DNA damage and apoptosis, leading to the suppression of proliferation, migration, and invasion activities. MNCT has been shown to inhibit tumour growth to a significant extent in mice. Furthermore, MNCT depletes GSH, suppresses P-gp expression, and upregulates tumour suppressor protein phosphatase and tensin homologue (PTEN) expression, resulting in reduced DNA damage repair and reversal of cisplatin resistance [142].

Recently, Wang et al. (2023) demonstrated the synergistic potential of the PARP inhibitor niraparib and DOX against ovarian cancer. Both drugs are encapsulated in highly stable liposomes containing folate to target FRα overexpressed on the surface of ovarian cancer cells [144]. Using this approach, the targeted delivery of niraparib and DOX can be achieved via increased receptor-mediated endocytosis. The synergistic actions of niraparib and DOX are dependent on both the molar ratio and the cell line. Notably, a 10:1 molar ratio exerted consistent synergistic or additive effects on all tested HR-deficient cell lines and conversely antagonistic effects in cell lines with HR proficiency (PEO4 cells). Based on these results, the group suggested that combinations of DOX and PARPis, in general, may be inappropriate for patients with HR-proficient tumours. Nanoparticle-based combined drug delivery is one of the only methods for achieving ratiometric combinations at diseased sites in vivo.

Self-assembled nanostructures generated with BSA and gallic acid-containing olaparib in conjunction with gallium (Ga) (III) (olaparib-Ga) were initially described by Li et al. (2022) [140] and further characterised by the group (Li et al., 2023). These nanoparticles administered in combination with cisplatin or carboplatin could successfully reverse PARPi resistance in platinum-resistant A2780 cis and SKOV3-cis ovarian cancer cells as well as SKOV3-cis tumour models. The primary mechanisms of action of Ga^3+^ in cancer therapy have been ascribed to its resemblance to Fe^3+^, resulting in Fe deprivation and subsequent inhibition of the activities of several enzymes and mitochondria-dependent apoptosis. The results showed that the combinations induced DNA damage, followed by the activation of ataxia telangiectasia mutated (ATM) and ataxia telangiectasia and Rad3-related (ATR) proteins, which subsequently activated checkpoint kinases 1 and 2 (Chk1 and Chk2). Combined treatment with olaparib-Ga and cisplatin or carboplatin led to significant suppression of tumour growth compared with that caused by single drug or control treatment in platinum-resistant OC female BALB/c nude mice [151].

Nanoparticles may also be helpful when used for the delivery of a combination of PARPi and WEE1. WEE1 kinase is a crucial regulator of the G2/M checkpoint, which provides additional time to repair damaged DNA before entry into mitosis. Earlier studies have indicated that WEE1 is overexpressed in OC patients and is correlated with poor prognosis. The combined effect of the two inhibitors (PARPi and WEE1) led to a significant increase in treatment efficacy. PARPi has been shown to induce DNA damage, resulting in cell cycle arrest in the G2 phase for DNA repair. At this point, WEE1 overrides olaparib-induced G2 blockade, leading to premature mitotic entry with massive DNA damage and consequent cell death [152]. Coadministration of WEE1 inhibitors (e.g., adavosertib) and PARP inhibitors (e.g., olaparib) is reported to be effective in inhibiting ovarian tumour growth regardless of BRCA mutation status but poorly tolerated when the compounds are used in their free form due to common overlapping toxic effects [153,154]. To overcome this problem, mesoporous melanin-like polydopamine (MPDA) NPs with good biocompatibility and high drug loading capacity for targeted co-delivery of Ada and Ola were used to treat ovarian cancer [155]. The mesoporous structure of MPDA nanoparticles allows the loading of both adavosertib and olaparib on the surface through π-π stacking and/or hydrogen binding. These NPs induced apoptosis in OVCAR8 and SKOV3 OC cells and significantly enhanced the synergistic activity of adavosertib and olaparib in OVCAR8 tumour-bearing BALB/c nude mice and patient-derived xenograft mice models with fresh OC samples, while minimising undesirable toxic side effects [155].

## 5. Limitations and Future Perspectives

Nanomedicine improves the solubility of drugs, controls drug proportions, targets drugs to the tumour site, reduces toxicity, and prevents multidrug resistance, which increases the prognosis of ovarian cancer patients. Combination delivery of a chemo drug and gene therapy using nanoparticles minimises the accumulation of the compounds in the normal tissues and allows the combined therapy to be co-delivered to the cancer cell. Gene therapy involving liposomal delivery has demonstrated encouraging results; however, limitations must be considered. MiRNA is susceptible to off-target effects. It has been reported that off-target gene silencing can lead to neurotoxicity and immunotoxicity and may reduce its therapeutic effects [156].

Drug delivery nanosystems are capable of overcoming these issues, potentiating the effect of classic chemotherapy, new chemotherapy based on replication stress inhibitors like PARP inhibitors or gene therapy through targeted and selective delivery to affected cells. The design of these nanosystems has used different lipids to produce liposomes (anionic, cationic, and neutral). Lipids with cationic headgroups have better features for gene delivery like easy complexation between lipids and genes by electrostatic interactions, interactions with cell membranes through electrostatic contact, and the ability to escape from the endosomes by their interaction and fusion with the endosome membrane [157].

Numerous studies have confirmed the utility of NPs in ovarian cancer therapy. NPs reduce the quantity of free drugs in circulation, thereby limiting passage to non-target tissues and cells, which may aid in overcoming the drug resistance of tumours. NCT03161132, a Grupo Español de Investigación en Cáncer de Ovario (GEICO) phase II trial/ROLANDO study, evaluated the efficacy of a combination of olaparib and PLD in patients with platinum-resistant HGSOC or endometroid ovarian cancer, regardless of their *BRCA* status. Among the 31 patients enrolled in the study, 16% contained *BRCA* mutations. Patients were initially administered six cycles of 300 mg olaparib twice daily and IV PLD 40 mg/m^2^ every 28 days, followed by maintenance therapy with 300 mg olaparib twice a day until progression or toxicity occurred. Owing to toxicity, the initial dose of PLD was further reduced to 30 mg/m^2^. The progression-free survival rate at 6 months was 47%, and the overall disease control rate was 77%, supporting the suitability of the olaparib-PLD (30 mg/m^2^) combination for development as a possible therapeutic alternative for ovarian cancer resistant to platinum-based regimens regardless of the *BRCA* mutation status [158]. In a clinical trial of EP0057 (CRLX101) (NCT02389985), the efficacy of a nanoparticle drug conjugate of a cyclodextrin-based polymer backbone loaded with camptothecin (CPT) in combination with PTX was examined in patients with epithelial ovarian cancer. EP0057 (CRLX101) exhibited slow clearance, high plasma drug retention, and controlled slow release of CPT [159], although the overall response rate was not significantly different from the control rate. These collective findings support the superiority of NPs over free drugs. Other clinical trials using different nanoformulations for ovarian cancer treatment are shown in Table 2.

## 6. Conclusions

This review discusses the possibilities of classical chemotherapy, gene therapy, or PARP inhibitors in different nanocarriers used for OC treatment. Moreover, it focuses on targeted therapy of OC, especially using ligands targeting HER2, FR, CD44, or VEGF receptors. One of the latest achievements in ovarian cancer therapy is the concept of synthetic lethality and, consequently, the development of replicative stress inhibitors, including PARPis. PARPi compounds exhibit greater efficacy when combined with other compounds for the treatment of *BRCA*-mutated and wild-type cancers. Here, we have reviewed several nanotechnology approaches to produce nanomedicines of PARP inhibitors and their therapeutic uses. In most of these treatments, nanomedicines of PARP inhibitors have shown superiority over the PARP inhibitors as single agents. To address PARP resistance and poor bioavailability, the concept of co-encapsulation of two or more drugs has been explored. Moreover, combination therapies of PARP inhibitors with gene therapy or other chemotherapy drugs would require lower doses of drugs, thus providing safer and tolerable treatment. The encapsulated single- and dual-drug therapies based on PARPis reduced tumour burden and metastasis more effectively over time than the corresponding free drug versions [146]. The greater therapeutic efficacy of encapsulated drugs is mainly based on selective targeting by specific ligands. An additional coating of these nanoparticles with an active targeted ligand may significantly reduce the risk of off-target delivery and increase internalisation by cancer cells. These collective features could limit the adverse effects of drugs and markedly improve the therapeutic responses of patients with ovarian cancer. In OC therapy based on ligands, the level of the target receptors, the orientation of the ligands on the surface of the nanoparticle, and the density of the ligands in relation to the selected type of receptor are of great importance.

Despite the pronounced advantages of nanomedicine in the combined treatment of ovarian cancer compared to other methods of traditional therapy, the number of nanomedicines used clinically is still too low, especially for functionalised nanocarriers. Targeted genetic testing and a broader understanding of pathophysiological differences in individual patients will continue to lead to the development of nanomedicine towards personalised ovarian cancer therapy.

## Figures and Tables

**Table 2:** Clinical trials using nanoformulations in ovarian cancer treatment.

Compound	Nanoparticle	Type of Ovarian Cancer	Phase	Clinical Trial Number
Lurtotecan	liposome	Advanced or recurrent epithelial OC	2	NCT00010179 NCT00046800
Topotecan	liposome	Advanced solid tumours that have relapsed, are refractory to standard therapy, or for whom there is no standard therapy available	1	NCT00765973
Irinotecan and bevacizumab	liposomal irinotecan as sucrosofate	Platinum-resistant, recurrent or refractory OC	2	NCT04753216
Mitoxantrone hydrochloride	liposome	Platinum-resistant or platinum refractory relapsed OC	1	NCT04718376
Abraxane and carboplatin	paclitaxel formulated as an albumin-bound nanoparticle	Platinum-sensitive OC	2	NCT00466986
Abraxane and bevacizumab	paclitaxel formulated as an albumin-bound nanoparticle	Recurrent, platinum-resistant epithelial OC	2	NCT00407563
EP0057 (CRLX101) and bevacizumab	CPT formulated as a cyclodextrin-based nanoparticle	Recurrent, platinum-resistant OC	2	NCT01652079
EP0057 (CRLX101) and paclitaxel	CPT formulated as a cyclodextrin-based nanoparticle	Recurrent or persistent epithelial OC	1/2	NCT02389985 [159]
EP0057 (CRLX101) and olaparib	CPT formulated as a cyclodextrin-based nanoparticle	Platinum-resistant OC or advanced OC who have received at least one prior line of platinum-based chemotherapy followed by a PARP inhibitor	2	NCT04669002
EGEN-001	PEG-PEI-cholesterol Lipopolymer-encased IL-12 DNA Plasmid Vector GEN-1	Recurrent epithelial OC	2	NCT01118052
ELU- FRα-1	C’Dot drug conjugate (CDC), consisting of payloads (exatecans) and targeting moieties (folic acid analogues) covalently bound by linkers to the C’Dot particle carrier	Tumours overexpressing FRα	1/2	NCT05001282
CPC634	Docetaxel entrapped in CriPec nanoparticles	Platinum-resistant OC	2	NCT03742713 [160]
Apatinib and paclitaxel	Apatinib, an antiangiogenic agent targeting vascular endothelial growth factor receptor (VEGFR2) and albumin-bound paclitaxel	Recurrent, platinum-resistant OC	2	NCT03942068
Paclitaxel	Paclitaxel formulated as an albumin-stabilised nanoparticle	Recurrent or persistent platinum-resistant OC	2	NCT00499252
Paclical	Paclitaxel (micellar) nanoparticles	Epithelial ovarian cancer	3	NCT00989131
Rapamycin	nanoparticle albumin-bound rapamycin	Recurrent OC, stage III/IV	1	NCT02646319

## References

[1] Saorin A., Saorin G., Duzagac F., Parisse P., Cao N., Corona G., Cavarzerani E., Rizzolio F. (2024). Microfluidic production of amiodarone loaded nanoparticles and application in drug repositioning in ovarian cancer. Sci. Rep..

[2] Momenimovahed Z., Tiznobaik A., Taheri S., Salehiniya H. (2019). Ovarian cancer in the world: Epidemiology and risk factors. Int. J. Womens Health.

[3] Turkoglu O., Zeb A., Graham S., Szyperski T., Szender J.B., Odunsi K., Bahado-Singh R. (2016). Metabolomics of biomarker discovery in ovarian cancer: A systematic review of the current literature. Metabolomics.

[4] Cortez A.J., Tudrej P., Kujawa K.A., Lisowska K.M. (2018). Advances in ovarian cancer therapy. Cancer Chemother. Pharmacol..

[5] Walton J.B., Farquharson M., Mason S., Port J., Kruspig B., Dowson S., Stevenson D., Murphy D., Matzuk M., Kim J. (2017). CRISPR/Cas9-derived models of ovarian high grade serous carcinoma targeting Brca1, Pten and Nf1, and correlation with platinum sensitivity. Sci. Rep..

[6] Konstantinopoulos P.A., Ceccaldi R., Shapiro G.I., D’Andrea A.D. (2015). Homologous Recombination Deficiency: Exploiting the Fundamental Vulnerability of Ovarian Cancer. Cancer Discov..

[7] Miller R.E., Leary A., Scott C.L., Serra V., Lord C.J., Bowtell D., Chang D.K., Garsed D.W., Jonkers J., Ledermann J.A. (2020). ESMO recommendations on predictive biomarker testing for homologous recombination deficiency and PARP inhibitor benefit in ovarian cancer. Ann. Oncol..

[8] Ashworth A. (2008). A synthetic lethal therapeutic approach: Poly(ADP) ribose polymerase inhibitors for the treatment of cancers deficient in DNA double-strand break repair. J. Clin. Oncol..

[9] Hockings H., Miller R.E. (2023). The role of PARP inhibitor combination therapy in ovarian cancer. Ther. Adv. Med. Oncol..

[10] Wu W., Pu Y., Shi J. (2022). Nanomedicine-enabled chemotherapy-based synergetic cancer treatments. J. Nanobiotechnol..

[11] Pathade A.D., Kommineni N., Bulbake U., Thummar M.M., Samanthula G., Khan W. (2019). Preparation and Comparison of Oral Bioavailability for Different Nano-formulations of Olaparib. AAPS PharmSciTech.

[12] Rai S., Singh N., Bhattacharya S. (2022). Concepts on Smart Nano-Based Drug Delivery System. Recent. Pat. Nanotechnol..

[13] Seca C., Ferraresi A., Phadngam S., Vidoni C., Isidoro C. (2019). Autophagy-dependent toxicity of amino-functionalized nanoparticles in ovarian cancer cells. J. Mater. Chem. B.

[14] Estanqueiro M., Amaral M.H., Conceição J., Sousa Lobo J.M. (2015). Nanotechnological carriers for cancer chemotherapy: The state of the art. Colloids Surf. B Biointerfaces.

[15] Matsumura Y., Maeda H. (1986). A new concept for macromolecular therapeutics in cancer chemotherapy: Mechanism of tumoritropic accumulation of proteins and the antitumor agent smancs. Cancer Res..

[16] O’Brien M.E.R., Wigler N., Inbar M., Rosso R., Grischke E., Santoro A., Catane R., Kieback D.G., Tomczak P., Ackland S.P. (2004). Reduced cardiotoxicity and comparable efficacy in a phase IIItrial of pegylated liposomal doxorubicin HCl(CAELYX™/Doxil^®^) versus conventional doxorubicin forfirst-line treatment of metastatic breast cancer. Ann. Oncol..

[17] Lee J.H., Yeo Y. (2015). Controlled drug release from pharmaceutical nanocarriers. Chem. Eng. Sci..

[18] Sindhwani S., Syed A.M., Ngai J., Kingston B.R., Maiorino L., Rothschild J., MacMillan P., Zhang Y., Rajesh N.U., Hoang T. (2020). The entry of nanoparticles into solid tumours. Nat. Mater..

[19] Lu Q., Kou D., Lou S., Ashrafizadeh M., Aref A.R., Canadas I., Tian Y., Niu X., Wang Y., Torabian P. (2024). Nanoparticles in tumor microenvironment remodeling and cancer immunotherapy. J. Hematol. Oncol..

[20] Qiu N., Liu X., Zhong Y., Zhou Z., Piao Y., Miao L., Zhang Q., Tang J., Huang L., Shen Y. (2016). Esterase-Activated Charge-Reversal Polymer for Fibroblast-Exempt Cancer Gene Therapy. Adv. Mater..

[21] Luo Z., Dai Y., Gao H. (2019). Development and application of hyaluronic acid in tumor targeting drug delivery. Acta Pharm. Sin. B.

[22] Prabhakar U., Maeda H., Jain R.K., Sevick-Muraca E.M., Zamboni W., Farokhzad O.C., Barry S.T., Gabizon A., Grodzinski P., Blakey D.C. (2013). Challenges and Key Considerations of the Enhanced Permeability and Retention Effect for Nanomedicine Drug Delivery in Oncology. Cancer Res..

[23] Nakamura Y., Mochida A., Choyke P.L., Kobayashi H. (2016). Nanodrug Delivery: Is the Enhanced Permeability and Retention Effect Sufficient for Curing Cancer?. Bioconjug. Chem..

[24] Dinarvand R., Sepehri N., Manouchehri S., Rouhani H., Atyabi F. (2011). Polylactide-co-glycolide nanoparticles for controlled delivery of anticancer agents. Int. J. Nanomed..

[25] Domínguez-Ríos R., Sánchez-Ramírez D.R., Ruiz-Saray K., Oceguera-Basurto P.E., Almada M., Juárez J., Zepeda-Moreno A., del Toro-Arreola A., Topete A., Daneri-Navarro A. (2019). Cisplatin-loaded PLGA nanoparticles for HER2 targeted ovarian cancer therapy. Colloids Surf. B Biointerfaces.

[26] Shapira A., Livney Y.D., Broxterman H.J., Assaraf Y.G. (2011). Nanomedicine for targeted cancer therapy: Towards the overcoming of drug resistance. Drug Resist. Updates.

[27] Kumari A., Singla R., Guliani A., Yadav S.K. (2014). Nanoencapsulation for drug delivery. EXCLI J..

[28] Mitchell M.J., Billingsley M.M., Haley R.M., Wechsler M.E., Peppas N.A., Langer R. (2020). Engineering precision nanoparticles for drug delivery. Nat. Rev. Drug Discov..

[29] Aggarwal R., Targhotra M., Kumar B., Sahoo P.K., Chauhan M.K. (2019). Polyplex: A Promising Gene Delivery System. Int. J. Pharm. Sci. Nanotechnol..

[30] Sercombe L., Veerati T., Moheimani F., Wu S.Y., Sood A.K., Hua S. (2015). Advances and Challenges of Liposome Assisted Drug Delivery. Front. Pharmacol..

[31] Nsairat H., Khater D., Sayed U., Odeh F., Al Bawab A., Alshaer W. (2022). Liposomes: Structure, composition, types, and clinical applications. Heliyon.

[32] Liu C., Zhang L., Zhu W., Guo R., Sun H., Chen X., Deng N. (2020). Barriers and Strategies of Cationic Liposomes for Cancer Gene Therapy. Mol. Ther. Methods Clin. Dev..

[33] Drakopoulou E., Anagnou N.P., Pappa K.I. (2022). Gene Therapy for Malignant and Benign Gynaecological Disorders: A Systematic Review of an Emerging Success Story. Cancers.

[34] Cai L., Xu X., Chen W. (2022). The Current State of the Art in PARP Inhibitor-Based Delivery Nanosystems. Pharmaceutics.

[35] Barenholz Y. (2012). Doxil^®^—The first FDA-approved nano-drug: Lessons learned. J. Control. Release.

[36] Pisano C., Cecere S.C., Di Napoli M., Cavaliere C., Tambaro R., Facchini G., Scaffa C., Losito S., Pizzolorusso A., Pignata S. (2013). Clinical Trials with Pegylated Liposomal Doxorubicin in the Treatment of Ovarian Cancer. J. Drug Deliv..

[37] Green A.E., Rose P.G. (2006). Pegylated liposomal doxorubicin in ovarian cancer. Int. J. Nanomed..

[38] Gabizon A., Shmeeda H., Barenholz Y. (2003). Pharmacokinetics of Pegylated Liposomal Doxorubicin. Clin. Pharmacokinet..

[39] Gibson J.-M., Alzghari S., Ahn C., Trantham H., La-Beck N.M. (2013). The Role of Pegylated Liposomal Doxorubicin in Ovarian Cancer: A Meta-Analysis of Randomized Clinical Trials. Oncologist.

[40] Tian K., Jia X., Zhao X., Liu P. (2017). Biocompatible Reduction and pH Dual-Responsive Core Cross-Linked Micelles Based on Multifunctional Amphiphilic Linear–Hyperbranched Copolymer for Controlled Anticancer Drug Delivery. Mol. Pharm..

[41] Keller B.-L., Lohmann C.A., Kyeremateng S.O., Fricker G. (2022). Synthesis and Characterization of Biodegradable Poly(butyl cyanoacrylate) for Drug Delivery Applications. Polymers.

[42] Kanaani L., Ebrahimi Far M., Kazemi S.M., Choupani E., Mazloumi Tabrizi M., Ebrahimi Shahmabadi H., Akbarzadeh Khiyavi A. (2017). General Characteristics and Cytotoxic Effects of Nano-Poly (Butyl Cyanoacrylate) Containing Carboplatin on Ovarian Cancer Cells. Asian Pac. J. Cancer Prev..

[43] Shen Y.A., Li W.H., Chen P.H., He C.L., Chang Y.H., Chuang C.M. (2015). Intraperitoneal delivery of a novel liposome-encapsulated paclitaxel redirects metabolic reprogramming and effectively inhibits cancer stem cells in Taxol((R))-resistant ovarian cancer. Am. J. Transl. Res..

[44] Liu Y., Ng Y., Toh M.R., Chiu G.N.C. (2015). Lipid-dendrimer hybrid nanosystem as a novel delivery system for paclitaxel to treat ovarian cancer. J. Control. Release.

[45] Kobayashi A., Yokoyama Y., Osawa Y., Miura R., Mizunuma H. (2015). Gene therapy for ovarian cancer using carbonyl reductase 1 DNA with a polyamidoamine dendrimer in mouse models. Cancer Gene Ther..

[46] Perez-Fidalgo J.A., Iglesias M., Bohn U., Calvo E., Garcia Y., Guerra E., Manso L., Santaballa A., Gonzalez-Martin A. (2019). GEICO1601-ROLANDO: A multicentric single arm Phase II clinical trial to evaluate the combination of olaparib and pegylated liposomal doxorubicin for platinum-resistant ovarian cancer. Future Sci. OA.

[47] Orlowski R.Z., Nagler A., Sonneveld P., Blade J., Hajek R., Spencer A., San Miguel J., Robak T., Dmoszynska A., Horvath N. (2007). Randomized phase III study of pegylated liposomal doxorubicin plus bortezomib compared with bortezomib alone in relapsed or refractory multiple myeloma: Combination therapy improves time to progression. J. Clin. Oncol..

[48] Schlüter B., Gerhards R., Strumberg D., Voigtmann R. (2010). Combined detection of Her2/neu gene amplification and protein overexpression in effusions from patients with breast and ovarian cancer. J. Cancer Res. Clin. Oncol..

[49] Ross J.S., Fletcher J.A., Linette G.P., Stec J., Clark E., Ayers M., Symmans W.F., Pusztai L., Bloom K.J. (2003). The HER-2/neu Gene and Protein in Breast Cancer 2003: Biomarker and Target of Therapy. Oncologist.

[50] Gutierrez C., Schiff R. (2011). HER2: Biology, detection, and clinical implications. Arch. Pathol. Lab. Med..

[51] Shah D., Osipo C. (2016). Cancer stem cells and HER2 positive breast cancer: The story so far. Genes Dis..

[52] Révillion F., Bonneterre J., Peyrat J.P. (1998). ERBB2 oncogene in human breast cancer and its clinical significance. Eur. J. Cancer.

[53] Stoecklein N.H., Luebke A.M., Erbersdobler A., Knoefel W.T., Schraut W., Verde P.E., Stern F., Scheunemann P., Peiper M., Eisenberger C.F. (2004). Copy Number of Chromosome 17 but Not HER2 Amplification Predicts Clinical Outcome of Patients with Pancreatic Ductal Adenocarcinoma. J. Clin. Oncol..

[54] English D.P., Roque D.M., Santin A.D. (2013). HER2 Expression Beyond Breast Cancer: Therapeutic Implications for Gynecologic Malignancies. Mol. Diagn. Ther..

[55] Lassus H. (2004). ERBB2 amplification is superior to protein expression status in predicting patient outcome in serous ovarian carcinoma. Gynecol. Oncol..

[56] Sun L.-Z., Luo H., Xu X., Ye M., Sheng B., Zhu X. (2018). The prognostic value of HER2 in ovarian cancer: A meta-analysis of observational studies. PLoS ONE.

[57] Meijer S.L., Wesseling J., Smit V.T., Nederlof P.M., Hooijer G.K., Ruijter H., Arends J.W., Kliffen M., van Gorp J.M., Sterk L. (2011). HER2 gene amplification in patients with breast cancer with equivocal IHC results. J. Clin. Pathol..

[58] Bookman M.A., Darcy K.M., Clarke-Pearson D., Boothby R.A., Horowitz I.R. (2003). Evaluation of monoclonal humanized anti-HER2 antibody, trastuzumab, in patients with recurrent or refractory ovarian or primary peritoneal carcinoma with overexpression of HER2: A phase II trial of the Gynecologic Oncology Group. J. Clin. Oncol..

[59] Gordon M.S., Matei D., Aghajanian C., Matulonis U.A., Brewer M., Fleming G.F., Hainsworth J.D., Garcia A.A., Pegram M.D., Schilder R.J. (2006). Clinical activity of pertuzumab (rhuMAb 2 C4), a HER dimerization inhibitor, in advanced ovarian cancer: Potential predictive relationship with tumor HER2 activation status. J. Clin. Oncol..

[60] Holm J., Hansen S.I. (2020). Characterization of soluble folate receptors (folate binding proteins) in humans. Biological roles and clinical potentials in infection and malignancy. Biochim. Biophys. Acta Proteins Proteom..

[61] Cheung A., Opzoomer J., Ilieva K.M., Gazinska P., Hoffmann R.M., Mirza H., Marlow R., Francesch-Domenech E., Fittall M., Dominguez Rodriguez D. (2018). Anti-Folate Receptor Alpha-Directed Antibody Therapies Restrict the Growth of Triple-negative Breast Cancer. Clin. Cancer Res..

[62] Kelemen L.E. (2006). The role of folate receptor alpha in cancer development, progression and treatment: Cause, consequence or innocent bystander?. Int. J. Cancer.

[63] Elnakat H., Ratnam M. (2004). Distribution, functionality and gene regulation of folate receptor isoforms: Implications in targeted therapy. Adv. Drug Deliv. Rev..

[64] Toffoli G., Cernigoi C., Russo A., Gallo A., Bagnoli M., Boiocchi M. (1997). Overexpression of folate binding protein in ovarian cancers. Int. J. Cancer.

[65] Boogerd L.S.F., Boonstra M.C., Beck A.-J., Charehbili A., Hoogstins C.E.S., Prevoo H.A.J.M., Singhal S., Low P.S., van de Velde C.J.H., Vahrmeijer A.L. (2016). Concordance of folate receptor-α expression between biopsy, primary tumor and metastasis in breast cancer and lung cancer patients. Oncotarget.

[66] O’Shannessy D.J., Somers E.B., Maltzman J., Smale R., Fu Y.S. (2012). Folate receptor alpha (FRA) expression in breast cancer: Identification of a new molecular subtype and association with triple negative disease. Springerplus.

[67] Bax H.J., Chauhan J., Stavraka C., Santaolalla A., Osborn G., Khiabany A., Grandits M., Lopez-Abente J., Palhares L., Chan Wah Hak C. (2023). Folate receptor alpha in ovarian cancer tissue and patient serum is associated with disease burden and treatment outcomes. Br. J. Cancer.

[68] Sun X., Zhao R., Zhao E., Wang Q., Lian W., Xiong J. (2022). Targeting CD44-positive ovarian cancers via engineered paclitaxel prodrug nanoparticles for enhanced chemotherapeutic efficacy. Biomed. Pharmacother..

[69] Zhong W., Pang L., Feng H., Dong H., Wang S., Cong H., Shen Y., Bing Y. (2020). Recent advantage of hyaluronic acid for anti-cancer application: A review of “3 S” transition approach. Carbohydr. Polym..

[70] Saman H., Raza S.S., Uddin S., Rasul K. (2020). Inducing Angiogenesis, a Key Step in Cancer Vascularization, and Treatment Approaches. Cancers.

[71] Liu Z.L., Chen H.H., Zheng L.L., Sun L.P., Shi L. (2023). Angiogenic signaling pathways and anti-angiogenic therapy for cancer. Signal Transduct. Target. Ther..

[72] Gulia M., Nishal S., Maddiboyina B., Dutt R., Kumar Desu P., Wadhwa R., Jhawat V. (2023). Physiological Pathway, diagnosis and nanotechnology based treatment strategies for ovarian Cancer: A review. Med. Omics.

[73] Mei C., Gong W., Wang X., Lv Y., Zhang Y., Wu S., Zhu C. (2023). Anti-angiogenic therapy in ovarian cancer: Current understandings and prospects of precision medicine. Front. Pharmacol..

[74] Lin W.-W., Cheng Y.-A., Li C.-C., Ho K.-W., Chen H.-J., Chen I.J., Huang B.-C., Liu H.-J., Lu Y.-C., Cheng C.-M. (2021). Enhancement of tumor tropism of mPEGylated nanoparticles by anti-mPEG bispecific antibody for ovarian cancer therapy. Sci. Rep..

[75] Son J.S., Chow R., Kim H., Lieu T., Xiao M., Kim S., Matuszewska K., Pereira M., Nguyen D.L., Petrik J. (2023). Liposomal delivery of gene therapy for ovarian cancer: A systematic review. Reprod. Biol. Endocrinol..

[76] Kotcherlakota R., Srinivasan D.J., Mukherjee S., Haroon M.M., Dar G.H., Venkatraman U., Patra C.R., Gopal V. (2017). Engineered fusion protein-loaded gold nanocarriers for targeted co-delivery of doxorubicin and erbB2-siRNA in human epidermal growth factor receptor-2+ ovarian cancer. J. Mater. Chem. B.

[77] Chen F., Ehlerding E.B., Cai W. (2014). Theranostic Nanoparticles. J. Nucl. Med..

[78] Stahl S., Graslund T., Eriksson Karlstrom A., Frejd F.Y., Nygren P.A., Lofblom J. (2017). Affibody Molecules in Biotechnological and Medical Applications. Trends Biotechnol..

[79] Tolmachev V., Orlova A. (2020). Affibody Molecules as Targeting Vectors for PET Imaging. Cancers.

[80] Satpathy M., Wang L., Zielinski R., Qian W., Lipowska M., Capala J., Lee G.Y., Xu H., Wang Y.A., Mao H. (2013). Active Targeting Using HER-2-Affibody-Conjugated Nanoparticles Enabled Sensitive and Specific Imaging of Orthotopic HER-2 Positive Ovarian Tumors. Small.

[81] Satpathy M., Wang L., Zielinski R.J., Qian W., Wang Y.A., Mohs A.M., Kairdolf B.A., Ji X., Capala J., Lipowska M. (2019). Targeted Drug Delivery and Image-Guided Therapy of Heterogeneous Ovarian Cancer Using HER2-Targeted Theranostic Nanoparticles. Theranostics.

[82] Maria B., Andrew H.S., Simon P.L. (2018). Human epidermal growth factor receptor targeted inhibitors for the treatment of ovarian cancer. Cancer Biol. Med..

[83] Vergote I., Armstrong D., Scambia G., Teneriello M., Sehouli J., Schweizer C., Weil S.C., Bamias A., Fujiwara K., Ochiai K. (2016). A Randomized, Double-Blind, Placebo-Controlled, Phase III Study to Assess Efficacy and Safety of Weekly Farletuzumab in Combination with Carboplatin and Taxane in Patients with Ovarian Cancer in First Platinum-Sensitive Relapse. J. Clin. Oncol..

[84] Moore K.N., Vergote I., Oaknin A., Colombo N., Banerjee S., Oza A., Pautier P., Malek K., Birrer M.J. (2018). FORWARD I: A Phase III study of mirvetuximab soravtansine versus chemotherapy in platinum-resistant ovarian cancer. Future Oncol..

[85] Chuan D., Mu M., Hou H., Zhao N., Li J., Tong A., Zou B., Chen H., Han B., Guo G. (2021). Folic acid-functionalized tea polyphenol as a tumor-targeting nano-drug delivery system. Mater. Des..

[86] Saifi M.A., Seal S., Godugu C. (2021). Nanoceria, the versatile nanoparticles: Promising biomedical applications. J. Control. Release.

[87] Hijaz M., Das S., Mert I., Gupta A., Al-Wahab Z., Tebbe C., Dar S., Chhina J., Giri S., Munkarah A. (2016). Folic acid tagged nanoceria as a novel therapeutic agent in ovarian cancer. BMC Cancer.

[88] Patra A., Satpathy S., Naik P.K., Kazi M., Hussain M.D. (2022). Folate receptor-targeted PLGA-PEG nanoparticles for enhancing the activity of genistein in ovarian cancer. Artif. Cells Nanomed. Biotechnol..

[89] Ak G., Yilmaz H., Gunes A., Hamarat Sanlier S. (2018). In vitro and in vivo evaluation of folate receptor-targeted a novel magnetic drug delivery system for ovarian cancer therapy. Artif. Cells Nanomed. Biotechnol..

[90] Girija A.R. (2019). Medical Applications of Polymer/Functionalized Nanoparticle Systems. Polymer Composites with Functionalized Nanoparticles.

[91] Zhu D., Liu F., Ma L., Liu D., Wang Z. (2013). Nanoparticle-Based Systems for T1-Weighted Magnetic Resonance Imaging Contrast Agents. Int. J. Mol. Sci..

[92] Lee N., Hyeon T. (2012). Designed synthesis of uniformly sized iron oxide nanoparticles for efficient magnetic resonance imaging contrast agents. Chem. Soc. Rev..

[93] Zhang H., Li J., Hu Y., Shen M., Shi X., Zhang G. (2016). Folic acid-targeted iron oxide nanoparticles as contrast agents for magnetic resonance imaging of human ovarian cancer. J. Ovarian Res..

[94] Ma W.W., Tolcher A., Perez C.A., Orr D., Hamilton E., Zhao Y., Murciano-Goroff Y., Anders C., Adams G.P., Reddick C.W. (2023). Abstract CT255: ELU-FRα-1: A study to evaluate ELU001 in patients with solid tumors that overexpress folate receptor alpha (FRα). Cancer Res..

[95] He Z.Y., Deng F., Wei X.W., Ma C.C., Luo M., Zhang P., Sang Y.X., Liang X., Liu L., Qin H.X. (2016). Ovarian cancer treatment with a tumor-targeting and gene expression-controllable lipoplex. Sci. Rep..

[96] Guan J.T., Li X.X., Peng D.W., Zhang W.M., Qu J., Lu F., D’Amato R.J., Chi Z.L. (2020). MicroRNA-18 a-5 p Administration Suppresses Retinal Neovascularization by Targeting FGF1 and HIF1 A. Front. Pharmacol..

[97] He Z.Y., Zhang Y.G., Yang Y.H., Ma C.C., Wang P., Du W., Li L., Xiang R., Song X.R., Zhao X. (2018). In Vivo Ovarian Cancer Gene Therapy Using CRISPR-Cas9. Hum. Gene Ther..

[98] Li Y., Gao Y., Zhang X., Guo H., Gao H. (2020). Nanoparticles in precision medicine for ovarian cancer: From chemotherapy to immunotherapy. Int. J. Pharm..

[99] Deiss-Yehiely E., Brucks S.D., Boehnke N., Pickering A.J., Kiessling L.L., Hammond P.T. (2022). Surface Presentation of Hyaluronic Acid Modulates Nanoparticle-Cell Association. Bioconjug. Chem..

[100] Edelman R., Assaraf Y.G., Slavkin A., Dolev T., Shahar T., Livney Y.D. (2019). Developing Body-Components-Based Theranostic Nanoparticles for Targeting Ovarian Cancer. Pharmaceutics.

[101] Zhao M.D., Li J.Q., Chen F.Y., Dong W., Wen L.J., Fei W.D., Zhang X., Yang P.L., Zhang X.M., Zheng C.H. (2019). Co-Delivery of Curcumin and Paclitaxel by “Core-Shell” Targeting Amphiphilic Copolymer to Reverse Resistance in the Treatment of Ovarian Cancer. Int. J. Nanomed..

[102] Yang X., Iyer A.K., Singh A., Choy E., Hornicek F.J., Amiji M.M., Duan Z. (2015). MDR1 siRNA loaded hyaluronic acid-based CD44 targeted nanoparticle systems circumvent paclitaxel resistance in ovarian cancer. Sci. Rep..

[103] Shah V., Taratula O., Garbuzenko O.B., Taratula O.R., Rodriguez-Rodriguez L., Minko T. (2013). Targeted nanomedicine for suppression of CD44 and simultaneous cell death induction in ovarian cancer: An optimal delivery of siRNA and anticancer drug. Clin. Cancer Res..

[104] Chen L., Luo J., Zhang J., Wang S., Sun Y., Liu Q., Cheng C. (2023). Dual Targeted Nanoparticles for the Codelivery of Doxorubicin and siRNA Cocktails to Overcome Ovarian Cancer Stem Cells. Int. J. Mol. Sci..

[105] Klotz D.M., Kuhlmann J.D., Link T., Goeckenjan M., Hofbauer L.C., Gobel A., Rachner T.D., Wimberger P. (2022). Clinical impact of soluble Neuropilin-1 in ovarian cancer patients and its association with its circulating ligands of the HGF/c-MET axis. Front. Oncol..

[106] Zou L., Song X., Yi T., Li S., Deng H., Chen X., Li Z., Bai Y., Zhong Q., Wei Y. (2013). Administration of PLGA nanoparticles carrying shRNA against focal adhesion kinase and CD44 results in enhanced antitumor effects against ovarian cancer. Cancer Gene Ther..

[107] Webb P.M., Jordan S.J. (2017). Epidemiology of epithelial ovarian cancer. Best Pract. Res. Clin. Obs. Gynaecol..

[108] Guo T., Zhu Y., Yue M., Wang F., Li Z., Lin M. (2022). The Therapeutic Effects of DDP/CD44-shRNA Nanoliposomes in AMF on Ovarian Cancer. Front. Oncol..

[109] Teleanu R.I., Chircov C., Grumezescu A.M., Teleanu D.M. (2019). Tumor Angiogenesis and Anti-Angiogenic Strategies for Cancer Treatment. J. Clin. Med..

[110] Garcia J., Hurwitz H.I., Sandler A.B., Miles D., Coleman R.L., Deurloo R., Chinot O.L. (2020). Bevacizumab (Avastin^®^) in cancer treatment: A review of 15 years of clinical experience and future outlook. Cancer Treat. Rev..

[111] Zhang Y., Guo J., Zhang X.-L., Li D.-P., Zhang T.-T., Gao F.-F., Liu N.-F., Sheng X.-G. (2015). Antibody fragment-armed mesoporous silica nanoparticles for the targeted delivery of bevacizumab in ovarian cancer cells. Int. J. Pharm..

[112] Giri S., Karakoti A., Graham R.P., Maguire J.L., Reilly C.M., Seal S., Rattan R., Shridhar V. (2013). Nanoceria: A rare-earth nanoparticle as a novel anti-angiogenic therapeutic agent in ovarian cancer. PLoS ONE.

[113] Engelberth S.A., Hempel N., Bergkvist M. (2014). Development of Nanoscale Approaches for Ovarian Cancer Therapeutics and Diagnostics. Crit. Rev. Oncog..

[114] Kohlberger M., Gadermaier G. (2021). SELEX: Critical factors and optimization strategies for successful aptamer selection. Biotechnol. Appl. Biochem..

[115] Chen Y., Xu M., Guo Y., Tu K., Wu W., Wang J., Tong X., Wu W., Qi L., Shi D. (2017). Targeted chimera delivery to ovarian cancer cells by heterogeneous gold magnetic nanoparticle. Nanotechnology.

[116] Green L.S., Jellinek D., Jenison R., Ostman A., Heldin C.H., Janjic N. (1996). Inhibitory DNA ligands to platelet-derived growth factor B-chain. Biochemistry.

[117] Zhao J., Tan W., Zheng J., Su Y., Cui M. (2022). Aptamer Nanomaterials for Ovarian Cancer Target Theranostics. Front. Bioeng. Biotechnol..

[118] Bhattacharya S., Anjum M.M., Patel K.K. (2022). Gemcitabine cationic polymeric nanoparticles against ovarian cancer: Formulation, characterization, and targeted drug delivery. Drug Deliv..

[119] Noorani L., Stenzel M., Liang R., Pourgholami M.H., Morris D.L. (2015). Albumin nanoparticles increase the anticancer efficacy of albendazole in ovarian cancer xenograft model. J. Nanobiotechnol..

[120] Yang S., Green A., Brown N., Robinson A., Senat M., Testino B., Dinulescu D.M., Sridhar S. (2023). Sustained delivery of PARP inhibitor Talazoparib for the treatment of BRCA-deficient ovarian cancer. Front. Oncol..

[121] Kim G., Ison G., McKee A.E., Zhang H., Tang S., Gwise T., Sridhara R., Lee E., Tzou A., Philip R. (2015). FDA Approval Summary: Olaparib Monotherapy in Patients with Deleterious Germline BRCA-Mutated Advanced Ovarian Cancer Treated with Three or More Lines of Chemotherapy. Clin. Cancer Res..

[122] Montemorano L., Lightfoot M., Bixel K. (2019). Role of Olaparib as Maintenance Treatment for Ovarian Cancer: The Evidence to Date. OncoTargets Ther..

[123] Arora S., Balasubramaniam S., Zhang H., Berman T., Narayan P., Suzman D., Bloomquist E., Tang S., Gong Y., Sridhara R. (2021). FDA Approval Summary: Olaparib Monotherapy or in Combination with Bevacizumab for the Maintenance Treatment of Patients with Advanced Ovarian Cancer. Oncologist.

[124] Moore K., Colombo N., Scambia G., Kim B.G., Oaknin A., Friedlander M., Lisyanskaya A., Floquet A., Leary A., Sonke G.S. (2018). Maintenance Olaparib in Patients with Newly Diagnosed Advanced Ovarian Cancer. N. Engl. J. Med..

[125] Robson M., Im S.A., Senkus E., Xu B., Domchek S.M., Masuda N., Delaloge S., Li W., Tung N., Armstrong A. (2017). Olaparib for Metastatic Breast Cancer in Patients with a Germline BRCA Mutation. N. Engl. J. Med..

[126] de Bono J., Mateo J., Fizazi K., Saad F., Shore N., Sandhu S., Chi K.N., Sartor O., Agarwal N., Olmos D. (2020). Olaparib for Metastatic Castration-Resistant Prostate Cancer. N. Engl. J. Med..

[127] Colombo I., Lheureux S., Oza A.M. (2018). Rucaparib: A novel PARP inhibitor for BRCA advanced ovarian cancer. Drug Des. Dev. Ther..

[128] Coleman R.L., Oza A.M., Lorusso D., Aghajanian C., Oaknin A., Dean A., Colombo N., Weberpals J.I., Clamp A., Scambia G. (2017). Rucaparib maintenance treatment for recurrent ovarian carcinoma after response to platinum therapy (ARIEL3): A randomised, double-blind, placebo-controlled, phase 3 trial. Lancet.

[129] Anscher M.S., Chang E., Gao X., Gong Y., Weinstock C., Bloomquist E., Adeniyi O., Charlab R., Zimmerman S., Serlemitsos-Day M. (2021). FDA Approval Summary: Rucaparib for the Treatment of Patients with Deleterious BRCA-Mutated Metastatic Castrate-Resistant Prostate Cancer. Oncologist.

[130] Rose M., Burgess J.T., O’Byrne K., Richard D.J., Bolderson E. (2020). PARP Inhibitors: Clinical Relevance, Mechanisms of Action and Tumor Resistance. Front. Cell Dev. Biol..

[131] O’Malley D.M., Krivak T.C., Kabil N., Munley J., Moore K.N. (2023). PARP Inhibitors in Ovarian Cancer: A Review. Target. Oncol..

[132] Liao H., Ji F., Helleday T., Ying S. (2018). Mechanisms for stalled replication fork stabilization: New targets for synthetic lethality strategies in cancer treatments. EMBO Rep..

[133] Patel M., Nowsheen S., Maraboyina S., Xia F. (2020). The role of poly(ADP-ribose) polymerase inhibitors in the treatment of cancer and methods to overcome resistance: A review. Cell Biosci..

[134] Hanzlikova H., Kalasova I., Demin A.A., Pennicott L.E., Cihlarova Z., Caldecott K.W. (2018). The Importance of Poly(ADP-Ribose) Polymerase as a Sensor of Unligated Okazaki Fragments during DNA Replication. Mol. Cell.

[135] Maya-Mendoza A., Moudry P., Merchut-Maya J.M., Lee M., Strauss R., Bartek J. (2018). High speed of fork progression induces DNA replication stress and genomic instability. Nature.

[136] Baldwin P., Ohman A.W., Tangutoori S., Dinulescu D.M., Sridhar S. (2018). Intraperitoneal delivery of NanoOlaparib for disseminated late-stage cancer treatment. Int. J. Nanomed..

[137] Corradetti B., Freile P., Pells S., Bagnaninchi P., Park J., Fahmy T.M., de Sousa P.A. (2012). Paracrine signalling events in embryonic stem cell renewal mediated by affinity targeted nanoparticles. Biomaterials.

[138] Magalhaes J.A., Arruda D.C., Baptista M.S., Tada D.B. (2021). Co-Encapsulation of Methylene Blue and PARP-Inhibitor into Poly(Lactic-Co-Glycolic Acid) Nanoparticles for Enhanced PDT of Cancer. Nanomaterials.

[139] Zuo Z., Zhou Z., Chang Y., Liu Y., Shen Y., Li Q., Zhang L. (2024). Ribonucleotide reductase M2 (RRM2): Regulation, function and targeting strategy in human cancer. Genes Dis..

[140] Li Y., Cen Y., Fang Y., Tang S., Li S., Ren Y., Zhang H., Lu W., Xu J. (2022). Breaking the Iron Homeostasis: A “Trojan Horse” Self-Assembled Nanodrug Sensitizes Homologous Recombination Proficient Ovarian Cancer Cells to PARP Inhibition. ACS Nano.

[141] Liu J., Hu F., Wu M., Tian L., Gong F., Zhong X., Chen M., Liu Z., Liu B. (2021). Bioorthogonal Coordination Polymer Nanoparticles with Aggregation-Induced Emission for Deep Tumor-Penetrating Radio- and Radiodynamic Therapy. Adv. Mater..

[142] Liu Y., Wang Y., Guan X., Wu Q., Zhang M., Cui P., Wang C., Chen X., Meng X., Ma T. (2023). Reversal of Cisplatin Resistance in Ovarian Cancer by the Multitargeted Nanodrug Delivery System Tf-Mn-MOF@Nira@CDDP. ACS Appl. Mater. Interfaces.

[143] Neufeld M.J., DuRoss A.N., Landry M.R., Winter H., Goforth A.M., Sun C. (2019). Co-delivery of PARP and PI3 K inhibitors by nanoscale metal-organic frameworks for enhanced tumor chemoradiation. Nano Res..

[144] Wang L., Evans J.C., Ahmed L., Allen C. (2023). Folate receptor targeted nanoparticles containing niraparib and doxorubicin as a potential candidate for the treatment of high grade serous ovarian cancer. Sci. Rep..

[145] Dasa S.S.K., Diakova G., Suzuki R., Mills A.M., Gutknecht M.F., Klibanov A.L., Slack-Davis J.K., Kelly K.A. (2018). Plectin-targeted liposomes enhance the therapeutic efficacy of a PARP inhibitor in the treatment of ovarian cancer. Theranostics.

[146] Mensah L.B., Morton S.W., Li J., Xiao H., Quadir M.A., Elias K.M., Penn E., Richson A.K., Ghoroghchian P.P., Liu J. (2019). Layer-by-layer nanoparticles for novel delivery of cisplatin and PARP inhibitors for platinum-based drug resistance therapy in ovarian cancer. Bioeng. Transl. Med..

[147] Juan A., Noblejas-López M.d.M., Bravo I., Arenas-Moreira M., Blasco-Navarro C., Clemente-Casares P., Lara-Sánchez A., Pandiella A., Alonso-Moreno C., Ocaña A. (2022). Enhanced Antitumoral Activity of Encapsulated BET Inhibitors When Combined with PARP Inhibitors for the Treatment of Triple-Negative Breast and Ovarian Cancers. Cancers.

[148] Balmaña J., Tung N.M., Isakoff S.J., Graña B., Ryan P.D., Saura C., Lowe E.S., Frewer P., Winer E., Baselga J. (2014). Phase I trial of olaparib in combination with cisplatin for the treatment of patients with advanced breast, ovarian and other solid tumors. Ann. Oncol..

[149] Lee J.-M., Hays J.L., Chiou V.L., Annunziata C.M., Swisher E.M., Harrell M.I., Yu M., Gordon N., Sissung T.M., Ji J. (2017). Phase I/Ib study of olaparib and carboplatin in women with triple negative breast cancer. Oncotarget.

[150] Novohradsky V., Zajac J., Vrana O., Kasparkova J., Brabec V. (2018). Simultaneous delivery of olaparib and carboplatin in PEGylated liposomes imparts this drug combination hypersensitivity and selectivity for breast tumor cells. Oncotarget.

[151] Li Y., Cen Y., Tu M., Xiang Z., Tang S., Lu W., Zhang H., Xu J. (2023). Nanoengineered Gallium Ion Incorporated Formulation for Safe and Efficient Reversal of PARP Inhibition and Platinum Resistance in Ovarian Cancer. Research.

[152] Geenen J.J.J., Schellens J.H.M. (2017). Molecular Pathways: Targeting the Protein Kinase Wee1 in Cancer. Clin. Cancer Res..

[153] Fang Y., McGrail D.J., Sun C., Labrie M., Chen X., Zhang D., Ju Z., Vellano C.P., Lu Y., Li Y. (2019). Sequential Therapy with PARP and WEE1 Inhibitors Minimizes Toxicity while Maintaining Efficacy. Cancer Cell.

[154] Pilié P.G., Tang C., Mills G.B., Yap T.A. (2018). State-of-the-art strategies for targeting the DNA damage response in cancer. Nat. Rev. Clin. Oncol..

[155] Wang W., Xiong Y., Hu X., Lu F., Qin T., Zhang L., Guo E., Yang B., Fu Y., Hu D. (2023). Codelivery of adavosertib and olaparib by tumor-targeting nanoparticles for augmented efficacy and reduced toxicity. Acta Biomater..

[156] Chen Y., Gao D.Y., Huang L. (2015). In vivo delivery of miRNAs for cancer therapy: Challenges and strategies. Adv. Drug Deliv. Rev..

[157] Zheng Z., Li Z., Xu C., Guo B., Guo P. (2019). Folate-displaying exosome mediated cytosolic delivery of siRNA avoiding endosome trapping. J. Control. Release.

[158] Perez-Fidalgo J.A., Cortes A., Guerra E., Garcia Y., Iglesias M., Bohn Sarmiento U., Calvo Garcia E., Manso Sanchez L., Santaballa A., Oaknin A. (2021). Olaparib in combination with pegylated liposomal doxorubicin for platinum-resistant ovarian cancer regardless of BRCA status: A GEICO phase II trial (ROLANDO study). ESMO Open.

[159] Duska L.R., Krasner C.N., O’Malley D.M., Hays J.L., Modesitt S.C., Mathews C.A., Moore K.N., Thaker P.H., Miller A., Purdy C. (2021). A phase Ib/II and pharmacokinetic study of EP0057 (formerly CRLX101) in combination with weekly paclitaxel in patients with recurrent or persistent epithelial ovarian, fallopian tube, or primary peritoneal cancer. Gynecol. Oncol..

[160] Boere I., Vergote I., Hanssen R., Jalving M., Gennigens C., Ottevanger P., van de Wouw Y.J., Rijcken C.J.F., Mathijssen R.H.J., Ledermann J. (2023). CINOVA: A phase II study of CPC634 (nanoparticulate docetaxel) in patients with platinum resistant recurrent ovarian cancer. Int. J. Gynecol. Cancer.

